# A randomized, clinical trial investigating the use of a digital intervention to reduce delirium-associated agitation

**DOI:** 10.1038/s41746-023-00950-4

**Published:** 2023-10-30

**Authors:** Michelle Nicholas, Jessica Wittmann, Monica Norena, Marlena Ornowska, Steven Reynolds

**Affiliations:** 1 Intensive Care Unit, Fraser Health Authority C/O Royal Columbian Hospital, New Westminster, BC V3L 3W7 Canada; 2 Department of Biomedical Physiology and Kinesiology, Simon Fraser University, Burnaby, BC V5A 1S6 Canada; 3 Centre for Health Evaluation and Outcome Sciences, Vancouver, BC V6Z 1Y6 Canada

**Keywords:** Therapeutics, Randomized controlled trials

## Abstract

We aimed to determine if a novel digital therapeutic intervention could reduce agitation and unscheduled medication use in an adult delirious acute care population. Delirious participants were randomly allocated (1:1) to receive standard of care plus a single 4-hour exposure to the digital intervention “MindfulGarden”, which uses a screen-based delivery to display a nature landscape with dynamic adjustment of screen content in response to movement and sound or standard of care only. Between March 2021 and January 2022, 73 participants were enrolled with 70 completing the trial protocol and included in the final analysis with a mean age of 61 years and 68% being male (35 intervention, 35 control). Mean RASS was significantly lower across the 4-hour study period in the intervention arm 0.3 (0.85) vs 0.9 (0.93), *p* = 0.01. Exposure to a nature-based dynamic digital intervention showed benefits in agitation reduction.

## Introduction

Delirium is an acute neuropsychiatric disorder that affects up to 80% of critical care patients^1,2^. A recent systematic review estimated between $806–$24509 USD in attributed costs per case^3^. Common interventions such as mechanical ventilation, sedation, and surgery have all been associated with the development of delirium or cognitive dysfunction^1,4,5^. Evidence shows those with more severe cases of delirium are at a higher risk of death after hospital discharge, are more likely to develop dementia, and are more likely to have long-term deficits in cognition^6,7^.

Management of delirium-associated agitation is challenging. Delirious patients may experience hallucinations and become aggressive, posing a risk of physical harm to themselves and the healthcare staff^8,9^. Healthcare workers often resort to the use of chemical and physical restraints despite limited evidence and known risks^10–14^. Delirium care utilizing multi-component strategies such as cognitive stimulation and re-orientation^15–17^ have been shown to reduce delirium incidence^18–21^.

Recently, digital technology-based interventions are become more prevalent in the literature. For example, clinical trials such as E-CHOISIR have employed virtual reality (VR) to expose participants to natural environments in combination with music or hypnosis with outcomes related to anxiety and pain^22–25^. One of the challenges in applying VR to delirium management is the headset equipment itself, which is not feasible with actively agitated patients. As such, alternatives such as exposure to tablets or other screens have been investigated. Waszynski et al. used short 60–90 sec nature videos, or recordings of family messages on tablets in an RCT to determine the effect on measurable agitation in delirious in-patients. Both intervention arms showed a reduction in mean agitation scores which had returned to baseline 30 min after the intervention, showing promise in the benefits of nature imagery in de-escalating agitation^26^. The exact mechanisms as to why nature imagery has beneficial effects are unknown. Among other theories, it has been postulated that it has a positive impact on the hypothalamic-pituitary-adrenal axis and cortisol dysregulation^27^.

We aimed to determine if using a screen-based digital therapeutic intervention can reduce agitation and delirium, as well as reliance on unscheduled medication for its management. This was investigated by exposing patients to the “MindfulGarden” digital intervention, which displays nature-driven imagery delivery that is dynamically responsive to patient agitation in a randomized-controlled trial of 70 delirious patients recruited from the hospital setting.

## Results

### Participant recruitment and baseline characteristics

**Fig. 1:**
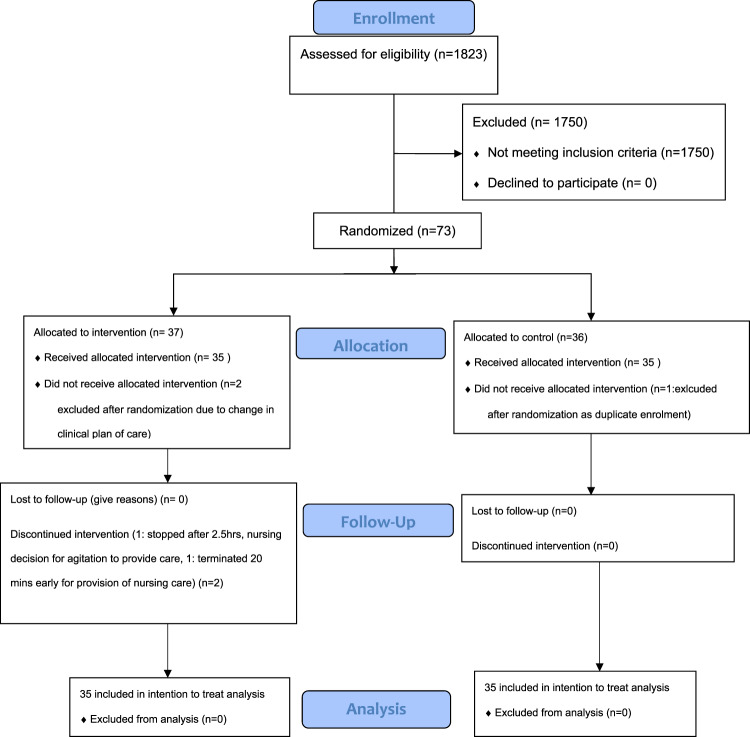
Flow diagram of the study population during each stage of study progress. The number of participants that proceeded or were lost through each of the enrolment, allocation, follow-up, and data analysis stages are indicated in brackets.

**Fig. 2:**
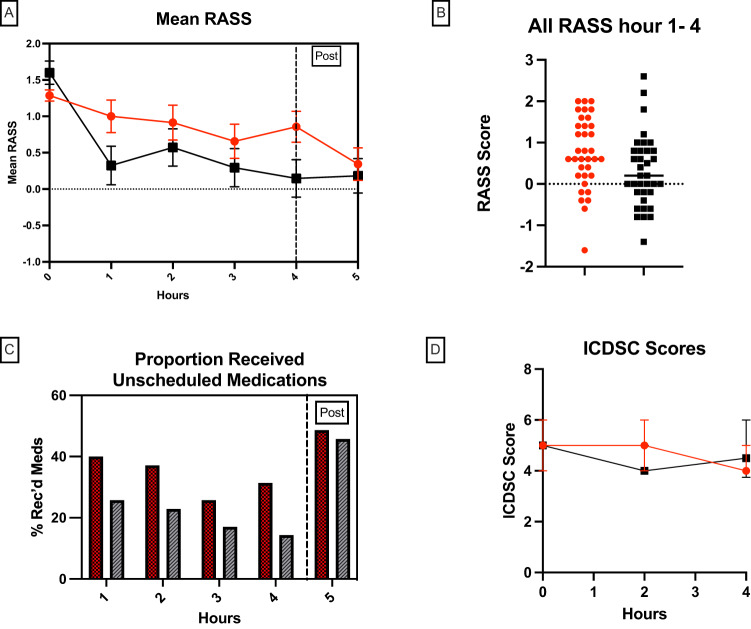
RASS, unscheduled medication, and ICDSC scoring between control and intervention groups. **A** Mean RASS (Richmond Agitation Sedation Scale) Mean RASS, error bars show the standard error of the mean (SEM) Hour 0 denotes pre-exposure scores, the dotted line at hour 4 shows the interventional period end. **B** Scatter estimation plot shows the distribution of all RASS scores between hours 1–4 during the study period, the line at group mean. This allows for the identification of outliers potentially driving the effect of the estimate. **C** All patients represented as a % in each study arm that received unscheduled medication by hour with “post” including in the 2-hours post-study completion. **D** *y* axis denotes the median ICDSC (Intensive care delirium screening checklist) with error bars showing 25th to 75th percentile. ICDSC is scored out of 8 with a score greater than or equal to 4 being diagnostic for delirium. Figure Legend: Red circles or bars refer to data collected for control participants, while black squares or bars represent data collected from those exposed to the intervention.

A total of 73 participants were recruited between March 16th, 2021, and January 5th, 2022, with 70 included in the final analysis (See Fig. 1). Three participants were excluded after randomization, of these two before the study start due to changes to the course of clinical care, and one that was a duplicate enrollment. Participants were recruited from critical care (*n* = 65) and high acuity cardiac telemetry wards (*n* = 5). See Table 1 for further details on patient demographics and characteristics. In the intervention arm, 2 participants did not complete the full 4-hour exposure, one ended 20 min early and one after 2.5 h of exposure due to nursing decisions for the necessary provision of care. All participants were analyzed according to intention to treat principles. (See Fig. 1) For logistical reasons one participant in the intervention arm did not have full data acquisition. Missed data points include RASS and ICDSC scores at hours 1,3, and 4. Mean RASS scores in the intervention and control arms were not significantly different at study initiation (1.6 (0.95) vs 1.2 (0.95) respectively, *p* = 0.27, via Kruskal–Wallis test). (See Fig. 2).

**Table 1:** Demographics and characteristics of included participants.

Demographic and medical characteristics:	Control (*n* = 35)	Intervention (*n* = 35)
Age: mean (range)	61.5 (20–89)	60.3 (19–86))
Sex: Male *n* (%)	21 (60.0)	27 (77.1)
BMI: mean (SD)	26.6 (4.83)	27.5 (6.02)
Renal replacement therapy *n* (%)	3 (8.6)	9 (25.7)
COPD *n* (%)	7 (20)	4 (11.4)
Underlying brain health condition (TBI, stroke, dementia) *n* (%)	11 (31.4)	14 (40)
Psychiatric history *n* (%) (eg depression, anxiety disorder, bi-polar)	11 (31.4)	12 (34.3)
Days since first delirium diagnosis: mean (SD)	3.9 (3.3)	4.5 (3.5)
Substance use history *n* (%)	8 (22.9)	9 (25.7)
APACHE IV score mean(SD)	37.1 (16.66)	43.7 (16.7)
Covid 19 positive *n* (%)	10 (28.6)	8 (22.9)
Diabetic *n* (%)	6 (17.1)	14 (40.0)
Mechanical ventilation at the time of study	7 (20.0%)	5 (14.3%)
Admission diagnosis:		
Trauma *n* (total %)	6 (17.1)	4 (11.4)
Traumatic brain injury *n* (%)	5 (14.3)	3 (8.6)
Neurological (non-traumatic) *n* (%)	4 (11.4)	6 (14.3)
Sepsis *n* (%)	2 (5.7)	3 (8.6)
Cardiovascular *n* (%)	11 (31.4)	11 (31.4)
Respiratory *n* (%)	10 (28.6)	9 (25.7)
Other *n* (%)	2 (5.7)	2 (5.7)

### Exposure to MindfulGarden significantly decreased the RASS score initially and maintained this throughout the duration of the intervention

At hour one post-intervention initiation, results of a *t* test show mean RASS scores were significantly decreased from baseline scores in the intervention group (−1.3 (2.11), *p* < 0.0001), but not the control group (−0.3 (1.34), *p* = 0.24). This corresponded with a significant difference in the proportion of participants showing a reduction of RASS at hour 1 with 24(70.6%) intervention vs 14 (40%) control (Chi-Square *p* = 0.01). This effect was maintained for the main outcome of mean RASS across the 4-hour study period which was significantly lower in the intervention arm (0.3 (0.85) vs 0.9 (0.93), *p* = 0.01, via Kruskal–Wallis test).

To account for potential confounding factors, we conducted a multivariate linear regression analysis to assess the difference in the average 4-hour RASS between the intervention and control groups during the study. The regression model was adjusted for key clinical variables obtained in the data collection process: age, gender, and pre-study RASS. Multivariate linear regression showed only the study arm (intervention allocation) was associated with an average reduction of 0.48 in the 4-hour RASS score over the duration of the study (Rate Ratio (95% CI) = −0.48, (−0.92 to −0.03), *p* = 0.04, see Table 2).

**Table 2:** Mean RASS and unscheduled medications regression analysis.

Outcome variable	Mean RASS RR (95% CI)	*p* value	Use of unscheduled medications (Y/N) OR (95%CI)	*p* value
Age	−0.01 (−0.01–0.01)	0.40	1 (0.97–1.03)	0.91
Female sex	0.11 (−0.36–0.58)	0.65	2.47 (0.76–8.05)	0.13
RASS pre-exposure	−0.13 (−0.43–0.17)	0.39		
Intervention vs control	−0.48 (−0.92—0.03)	0.04	0.36 (0.13–1.02)	0.06

The proportion of total RASS measurements during the 4-hour intervention period at 0 (calm and awake) or −1 (mildly drowsy) was significantly higher in the intervention group 63/137(46%) than in the control group 41/140 (29%), respectively (*p* = 0.004 via Chi-Square). Fifteen participants in each group achieved a RASS of zero at some point during the 4-hour study period. The proportion of RASS measures at 0 at any time over the 4-hour study period was higher in the intervention arm but this was not statistically significant, 32(23.4%) vs 26(18.6%) (*p* = 0.33 via Chi-Square test.

A-priori planned subgroup analyses of the effect of the intervention on specific groups were conducted using Kruskal–Wallis. Participants showed lower mean RASS scores in the intervention arm who were not mechanically ventilated at the time of the study (*p* = 0.003), had a diagnosis of delirium >24 h (*p* = 0.02), did not have a traumatic brain injury (TBI) (*p* = 0.02) and had a medical cause of admission (*p* < 0.0001). See Table 3 for a full breakdown of these results.

**Table 3:** Kruskal–Wallis subgroup analysis of RASS hours 1–4.

	Control	Intervention	*p* value
TBI-no	*n* = 30	*n* = 32	
Mean	0.8 (0.95)	0.4 (0.87)	0.02
Med	0.9 (0.5–1.5)	0.4 (−0.1–0.8)	
TBI-yes	*n* = 5	*n* = 3	
Mean	1.1 (0.91)	0.1 (0.63)	0.17
Med	0.8 (0.5–1.5)	0.0 (−0.5–0.8)	
MV-no	*n* = 28	*n* = 30	
Mean	0.9 (0.83)	0.3 (0.76)	0.003
Med	0.8 (0.5–1.5)	0.4 (0.0–0.8)	
MV-yes	*n* = 7	*n* = 5	
Mean	0.6 (1.31)	0.7 (1.36)	0.68
Med	1.0 (−0.5–1.8)	0.4 (0.0–0.8)	
Delirium>24 h-yes	*n* = 15	*n* = 11	
Mean	1.0 (0.78)	0.1 (0.74)	0.02
Med	0.9 (0.5–1.8)	0.4 (0.89)	
Medical admit	*n* = 22	*n* = 27	
Mean	1.1 (0.80)	0.1 (0.62)	<0.0001
Med	1.1 (0.5–1.8)	0.3 (−0.3–0.5)	
Surgical admit	*n* = 13	*n* = 8	
Mean	0.4 (1.02)	1.0 (1.17)	0.26
Med	0.5 (0.0–0.8)	1.0 (0.1–2.1)	

### Exposure to MindfulGarden significantly reduced the proportion of patients who received unscheduled medications for delirium and agitation management

For the secondary outcome of unscheduled medication use, a significant difference was shown in the proportion of participants receiving unscheduled medication throughout the four hours of the study period with 17 (48.6%) intervention vs 26 (74.3%) control, respectively (*p* = 0.03 via chi-square test). Multivariate logistic regression though, did not show the association between patient’s group and unscheduled medication use after adjusting for age and gender.

In the 2-hour post-trial period, the proportion receiving unscheduled medications was not significant between study arms, intervention 16 (45.7%) vs control 17 (48.6%),(RR 1.06 95% CI: 0.64–1.76 *p* = 0.8). Mean drug events per participant were not significantly different between the intervention and control groups, 1.26 (1.84) vs 1.69 (1.62) respectively, *p* = 0.30, via chi-square test.

### Exposure to MindfulGarden did not result in changes to ICDSC scores

Median delirium scores using ICDSC were similar pre-exposure in the intervention and the control groups, 5.0 (4.0–6.0) vs 5.0 (4.0–6.0) respectively, *p* = 0.62, via Kruskal–Wallis test. Similarly, they were not significantly different between the intervention and control groups at hour 2, Med (IQR) 4.0 (4.0–5.0) vs 5.0 (4.0–5.0), *p* = 0.66 and hour 4, 5.0 (4.0–6.0) vs 4.0 (4.0–5.0) respectively, *p* = 0.46, via Kruskal–Wallis test. In multivariate linear regression there was no significant association between patient’s group and either ICDSC scores at hour 2 nor hour 4 after adjusting for age and gender.

### Exposure to MindfulGarden did not result in a reduction of physical restraint use, self-inflicted and iatrogenic removal of medical devices, or time to any of these events

Use of physical restraints was common at study start, with 26 (74.3%) patients in the intervention arm using restraints versus 29 (82.9%) control in the control arm (*p* = 0.38 via Chi-Square test) and at 1-hour post-trial completion, 24 (69%) intervention vs 30 (86%) control (*p* = 0.09, via Chi-Square test). The proportion of participants reported to have an unplanned line or equipment removal (such as patient pulling out IVs or nasogastric tubes) was not significant between arms, 1 (2.9%) intervention vs 4 (11.4%) in the control (*p* = 0.36, via Fisher’s exact test) with 1 vs 5 total events respectively. No specific harms from the intervention were observed during the study period.

## Discussion

Our results show a significant reduction in agitation with exposure to the digital calming intervention that was maintained over the 4-hour study period. This reduction in RASS was achieved with fewer potentially toxic unscheduled medications. These findings are important as they set the foundation for digital therapeutics in delirious, hospitalized patients. What may be just as important as the decrease in mean RASS scores, is that 70% of participants exposed to the intervention had a reduction in RASS at hour one. A reduction in RASS is perhaps more significant than achieving a goal RASS of zero or −1. A reduction of more than 25% in patients requiring unscheduled medication use may have clinical benefits and is an important finding. The simultaneous reduction in RASS and unscheduled medication use for managing agitation gives more validity to the inference that patients were being calmed and distracted by the intervention. These reductions could have significant downstream benefits to patients by avoiding complications and reducing the burden on nursing staff. Although not studied, it may also reduce distressing aspects of the patient’s experience and may influence the course of delirium as physical and chemical restraints may in themselves contribute to delirium. While physical restraint use was high overall, this may be more reflective of having conducted the trial during the Covid 19 pandemic with significant strain on nursing resources.

The a priori planned subgroup analysis provides some insight as to which groups may benefit most from this intervention, although this must be interpreted with caution due to the small numbers in some subgroups. It seems reasonable that patients who were not intubated may derive the most benefit as the device could utilize vocalization as well as movement as markers of agitation. Interestingly, the intervention was more effective in patients without TBI, although there was a trend towards an effect in those with head injuries this may be a function of the small sample size. It is not clear why patients with a medical reason for admission were more responsive to the calming effects of the intervention although this too suffered from a small surgical sample size. A final subgroup that showed significantly more response to the intervention were those with a diagnosis of delirium of greater than 24 h. This group potentially had a more established pattern of agitation that was somewhat resistant to traditional non-pharmacological interventions. Likely transient delirium may not require the same degree of intensity of interventions that more established delirium does.

While agitation scores were reduced, measures of delirium were not, with no significant change in ICDSC scores over the study period. This may show that while the intervention is effective in reducing agitation, it was not effective at reversing or reducing measurable delirium. It is likely that the intervention distracts and calms the patient but does not change the underlying cause of delirium.

This trial’s most notable limitation is it being open-labeled and reliant on direct care nursing staff to score and report outcomes such as agitation scores. While this is part of the normal conduct of care, the inability to blind providers or outcome assessors to the intervention introduced possible bias. Similarly, a degree of Hawthorne effect may be present by staff self-modulating their response to patient agitation in the use of unscheduled medications knowing their practice was being observed. Indeed, the initial primary outcome was planned to be unscheduled drug use by the bedside nurses. However, this was felt too sensitive to potential bias and was changed to RASS scores within 4 months of study initiation and at 19% recruitment completed. This was before any data was accessed or analyzed. Although this change in the primary outcome should be considered a weakness, we felt it was reasonable as both were a priori planned outcome measures, they were already being gathered and the change was to what we felt provided a more rigorous primary outcome. Interestingly, both outcomes of RASS and unscheduled medication use showed a significant improvement with the intervention thus mitigating this potential weakness. The overall sample size is likely underpowered for some subgroup analyses. The interactive component of the intervention cannot be definitively shown to have a causative effect on the outcomes of interest. A comparison of a TV or intervention without the interactive component may be required to understand the effect more clearly, as well as the mechanism of action. While this study was completed in a predominantly critical care environment, it is reasonable to expect that the intervention would be effective, or even potentially amplified, in the general hospital population with a lower nurse-to-patient ratio. Ultimately the success of this intervention will depend upon its acceptance by nursing staff and the impact on patients. As an initial study, nursing and patient feedback was not systematically gathered but will be an important aspect of future evaluations.

There is a clear need for effective non-pharmacological interventions for the management of delirium. Our study provides the initial work demonstrating that interactive digital therapeutics are an effective non-pharmacological approach to managing agitated delirium. It may provide a strategy to reduce the burden of nursing care and improve resource utilization. Additional features added to this basic framework may be of benefit including noise inhibiting sound, scheduled re-orientation cues, and different interactive experiences tailored to personal preferences. Although this study was not powered to show clinical outcomes, there are potential benefits in terms of length of stay, morbidity, and economic burden to healthcare systems. Interactive digital therapeutics for delirium provide a novel adjunct to agitation management while potentially reducing the risk profile associated with traditional strategies. This novel non-pharmacological intervention may improve patient outcomes and reduce nursing burden although the optimal application of this new tool remains to be determined through future research.

## Methods

### Study design

We conducted a single-center, open-label randomized-controlled trial at a tertiary referral and trauma center (Royal Columbian Hospital) in New Westminster, Canada. The study continued until recruitment goals were met. Participants had to be admitted to intensive care, high acuity, and cardiac telemetry units. Eligible patients were randomized in a 1:1 ratio to either intervention plus standard of care, or standard of care only. (See Fig. 1). Harmonized ethics approval was obtained from Fraser Health Authority and Simon Fraser University regulatory ethics boards. This study was registered with ClinicalTrials.gov, NCT04652622.

### Participants and inclusion/exclusion criteria

Participants were adult inpatients with a RASS (Richmond Agitation Sedation Score) +1 or greater, for 2 assessments at least 1 h apart within the 24 h directly before study enrollment. The elevated RASS score had to be persisting at the time of enrollment. If the RASS score was not available, equivalent documentation of agitation related to delirium for participants admitted outside of critical care, and ICDSC (Intensive Care Delirium Screening Checklist) at the time of enrollment, or CAM (Confusion Assessment Method) positive screening was also considered^28–30^. Participants were also required to have at least 2 unscheduled medication events in the preceding 24 h and/or infusion of psycho-active medication (eg. Dexmedetomidine) for the management of delirium-associated agitation.

Participants were excluded if they had a planned procedure or test that precluded participation in the full 4-hour study session, were visually impaired, had significant uncontrolled pain, had RASS less than or equal to 0 at enrollment, refused participation by the responsible physician, or were enrolled in another research study which could impact on the outcomes of interest (as evaluated by the Principal Investigator). Participants were recruited with an approved waived consent process.

### Randomization

Eligible patients were randomized using a master randomization list generated by an independent statistician using block permutation (blocks of 2 or 4). Allocation was determined using sequentially numbered opaque envelopes previously filled by a non-research team member and opened after enrollment was confirmed.

### Blinding

Blinding to the intervention was not possible due to the nature of the intervention and the logistical constraints of the study.

### Procedures

**Fig. 3:**
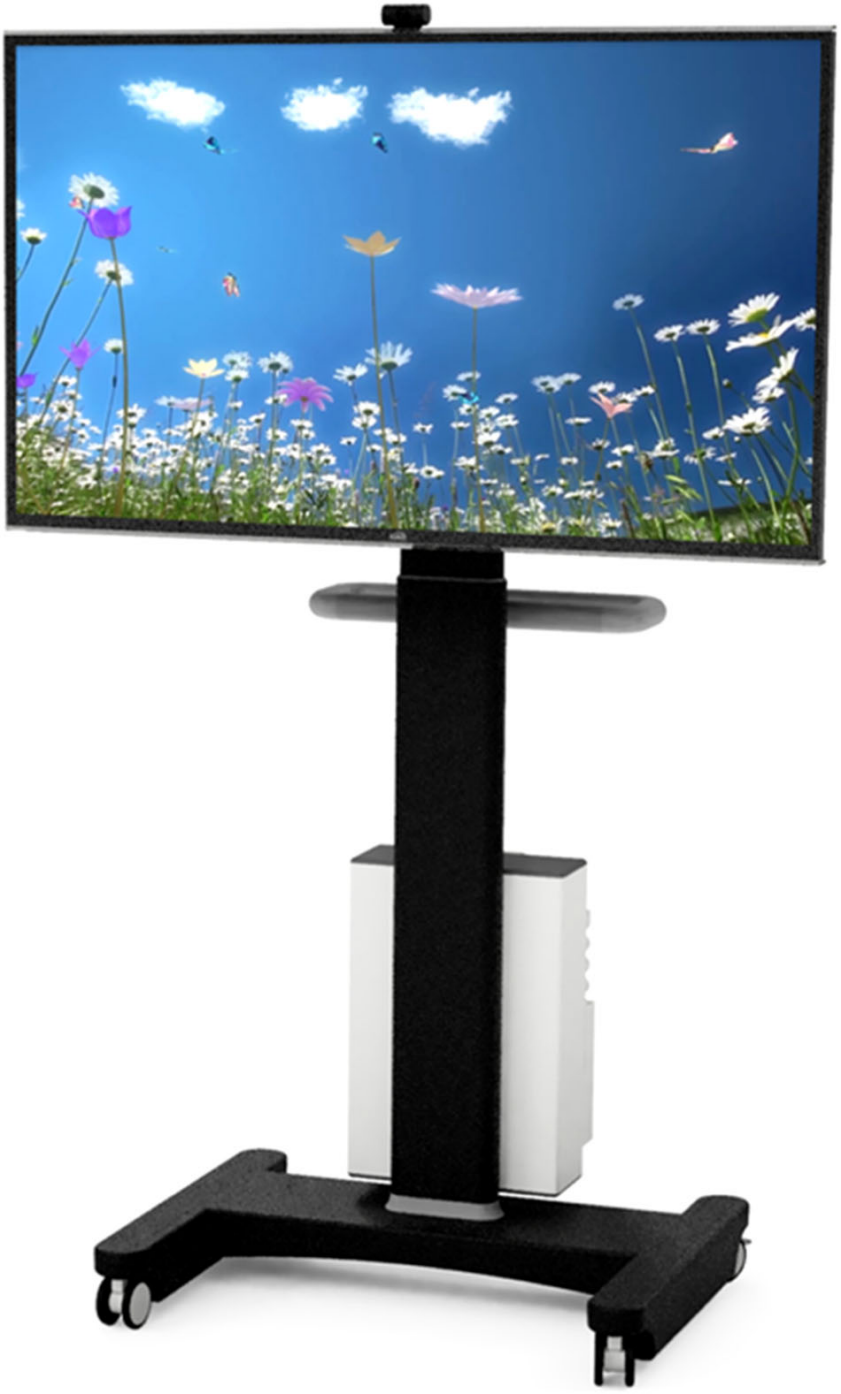
The MindfulGarden intervention. Used with permission of Mindful Garden Digital Health Inc. www.mindfulgarden.com.

The intervention, “MindfulGarden” utilizes a high-definition screen to present a pastoral scene layered with animations of butterflies and flowers blooming. It adjusts the volume of on-screen content in response to movement and sound production, which are surrogate markers of agitation (See Fig. 3 and Supplementary Fig. 1.0 and Note 1.0 for further details). The intervention only provided visual input to the patients, there was no auditory component produced.

For those randomized to the experimental arm, the display was placed near the foot of the bed for 4 consecutive hours. For the provision of care that required physical interaction with the participant, the device was placed in standby mode for 5-minute intervals. Mechanically ventilated patients had the microphone function disabled to avoid activation by the ventilator and its alarms.

The trial was conducted during daytime hours to allow the trial period to be completed within a single nursing shift where possible. Non-pharmacological distraction interventions were halted during the study period, such as other audio-visual interventions (TVs, tablets, or music) in both arms. Re-orientation by staff, use of whiteboards, clocks, family presence, repositioning, mobilization, physiotherapy, and general nursing care continued uninterrupted throughout the study period. The full protocol is available in Supplementary Note 2.

### Outcomes

The primary outcome was mean agitation (RASS) scores over the study period. RASS scores were measured pre-exposure, and every hour thereafter until one hour post the 4-hour intervention period. Secondary outcomes included the proportion of participants receiving unscheduled pharmacological interventions for the management of delirium-associated agitation during the 4-hour study period, delirium scores (ICDSC at intervention initiation, 2 h, and 4 h exposure time), the proportion of patients achieving target RASS of 0 or −1 (indicating awake and calm to mildly drowsy), the use of physical restraints, the incidence of self-inflicted harm, unplanned removal of lines, tubes or equipment by participants throughout the study period, including the time to event from the start of the study period of these events, as well as the proportion of participants receiving unscheduled pharmacological intervention in the 2-hours post-intervention.

### Data collection

For the outcomes of RASS and ICDSC scores, bedside nurses conducted assessments and documented scores on paper-based forms which were then collected by research staff. Nursing staff in critical care and high acuity areas used these scoring systems routinely in patient assessments. For participants enrolled in cardiac telemetry wards, observations were conducted by trained research personnel in collaboration with ward nurses.

### Statistical analysis

#### Sample size

Based on clinical experience in the ICU, it was anticipated that over a period of 4 hours, approximately 70% of agitated delirious patients would receive unscheduled medications for delirium. We anticipated the intervention would decrease this by a 50% relative reduction from 70% incidence to 35%. The required sample size was calculated to be 31 patients per arm, with a power of 80% and a significance level of 0.05. (www.clinicalc.com) We increased this slightly in recognition that it was an estimated effect size and is supported by previous literature^26,31^.

#### Statistical plan

Descriptive statistics are presented using mean (±SD) or median (IQR), with proportions being represented as the total number and percentage *n* (%). To assess differences between groups for various variables, the Kruskal-Wallis test was used. This test is an appropriate statistical test for variables that do not meet the normality assumption, as well as for variables that may meet normality assumptions. Proportions were tested using Chi-square or Fisher’s Exact tests. The primary outcome of RASS scores was further analyzed in a multivariate linear regression model with an arm as the primary exposure variable, adjusting for age, sex, and pre-exposure RASS score. Yes/No unscheduled drug administration was another outcome analyzed with multivariate logistic regression, again using the group as the exposure variable and adjusting for age and gender. All tests performed were two-sided.

An unscheduled drug event included the unscheduled use of antipsychotics, sedatives, or narcotics, and where participants were on continuous infusions of medications (e.g.: dexmedetomidine) a 20% increase in dose was considered an unscheduled event. A-priori subgroup analyses of mean RASS scores were planned to ascertain what may be the optimal target population for the intervention including the presence of TBI, mechanical ventilation at the time of the trial, delirium >24 h, and medical versus surgical cause of admission (Kruskal–Wallis, see Table 3). A *p* value < 0.05 was considered significant for all results. The main statistical analysis for the outcomes of RASS, regression, and subgroup analyses were conducted by an independent statistician using SAS Version 9.1. Secondary outcomes were analyzed using GraphPad Prism Version 9.4.1 (Table 4).

**Table 4:** ICDSC multivariate linear regression analysis.

Outcome variable	ICDSC hour 2 RR 95% CI	*p* value	ICDSC hour 4 RR 95% CI	*p* value
Age	0.02 (0.0, 0.04)	0.02	0.01 (−0.01, 0.03)	0.15
Female sex	0.3 (−0.45, 1.06)	0.43	−0.07 (−0.8, 0.66)	0.85
Intervention vs control	−0.1 (−0.81, 0.6)	0.77	0.29 (−0.39, 0.97)	0.39

The authors had sole responsibility for study design, data collection, data analysis, data interpretation, and writing of the report.

### Reporting summary

Further information on research design is available in the Nature Research Reporting Summary linked to this article.

### Supplementary information

Supplemental Materials

Reporting Summary

## Data Availability

Aggregate data analyzed in this study may be made available upon reasonable request by contacting the corresponding author via the e-mail address provided.
