# Supplementary material for: A randomized, clinical trial investigating the use of a digital intervention to reduce delirium-associated agitation
Source: NPJ Digit Med. 2023 Oct 30;6:202. doi: 10.1038/s41746-023-00950-4 (PMC10616287; doi:10.1038/s41746-023-00950-4)
Supplement: Supplementary file 1 — Supplemental Materials [file 41746_2023_950_MOESM1_ESM.pdf]

Supplementary Figure 1: The Mindful Garden Unit

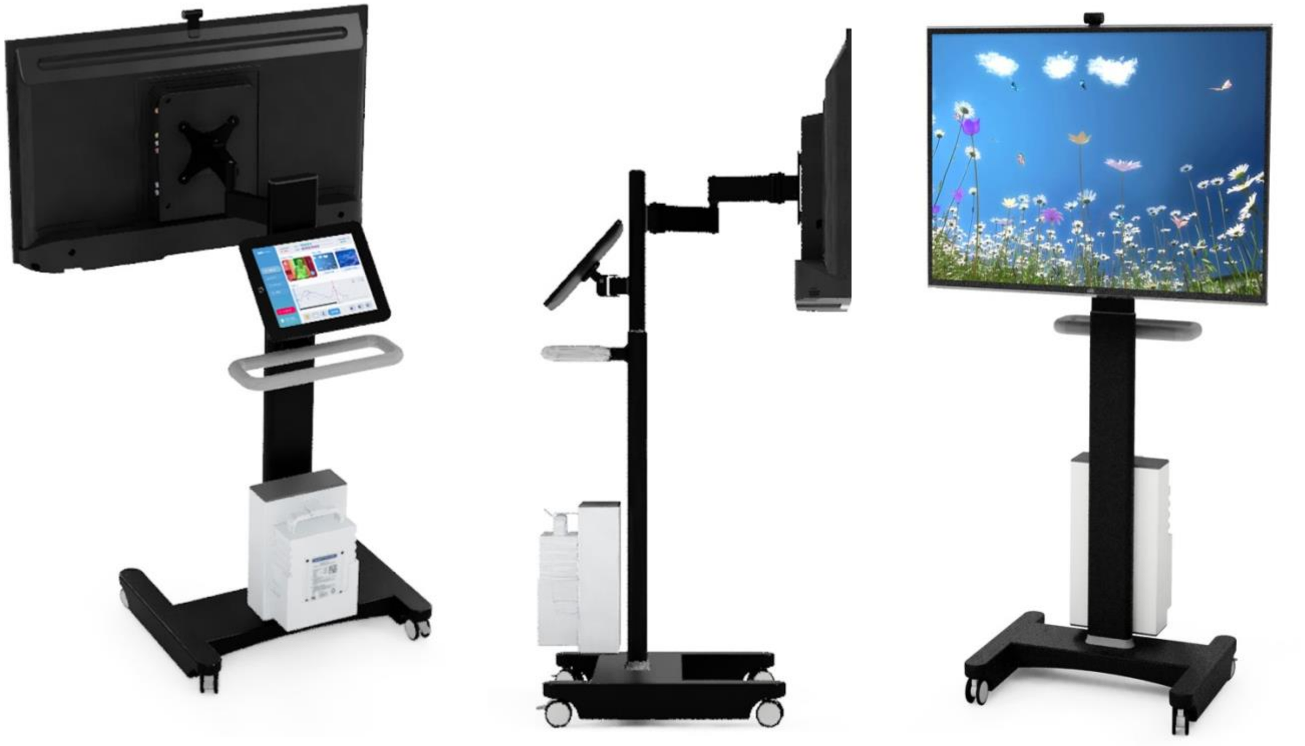

Supplementary Note 1: MindfulGarden Unit Details

Basic function: The unit is pre-commercial. It uses an attached camera and microphone to view the patient and measures sound production in decibels and fluctuations in movement using pixel density. This drives proprietary algorithms to control the on-screen content. The screen is mounted on an articulating arm to allow positional adjustment and a wheeled stand. The MindfulGarden unit utilizes a rechargeable medical-grade battery to allow for further ease of use. The unit does not physically attach to the patient.

High-Definition Digital Display: The screen displays a video of a meadow of flowers that is layered with animations of butterflies in flight and flowers that bloom and recede. The animations fluctuate in volume driven by the patient agitation measurement algorithms.
The animations move at a relaxed speed and are designed to provide a calming experience for the viewer. There is the capability to adjust the responsiveness, speed, and volume of animations; however, all settings were locked at default mid-range settings for this trial.
The on-screen experience can adjust the level of brightness according to the time of day to promote natural circadian rhythm. For this trial, all patients received the standard "daylight" settings.

Controls: A touchpad attached to the rear of the monitor allowed access to controls and to the standby feature utilized to freeze input for 5-minute intervals without adjusting the current on-screen content. The unit used an automatic restart that could be overridden and started by direct care staff if the provision of care or interaction took less than 5 minutes. The timer could be reactivated without limits. The touchscreen display used a digital readout to allow the user to ensure that the participant was captured within the camera range and measurement zone to limit extraneous activity from activating the intervention.

Sound: Sound input was deactivated for those receiving mechanical ventilation to avoid auditory activation from the ventilator and associated alarms. For the purposes of this study, the noise-masking soundtracks were not utilized to be able to determine the effect of the intervention more accurately as a visual therapy.

<u>Database</u>: Anonymized patient and session data is encrypted and logged to a secure database on the unit, providing dashboard analytics.

Security: All Wi-Fi and Bluetooth connectivity were disabled and recording functions were turned off for the purposes of this trial to ensure patient privacy and anonymity.

Supplementary Table 1: Use of physical restraints Y/N – Control Arm

Participant	Hour 0	Type	Hour 1	Type	Hour 2	Type	Hour 3	Type	Hour 4	Type	Hour 5	Type
1	y	1 X L W SOFT	y	2 X W SOFT, 1 X L SOFT	y	1 X L W SOFT	y	2 X W SOFT	n		Y	1 X L W SOFT
2	y	4 X SOFT	y	4 X SOFT	y	4 X SOFT	y	4 X SOFT	y	4 X SOFT	Y	4 X SOFT
3	y	2 X W SOFT	y	2 X W SOFT	y	2 X W SOFT	y	2 X W SOFT	y		y	2 X W SOFT
4	y	2 X W SOFT	y	2 X W SOFT	y	2 X W SOFT	y	2 X W SOFT	y	2 X W SOFT	y	2 X W SOFT
5	y	2 X W SOFT	y	2 X W SOFT	y	2 X W SOFT	y	2 X W SOFT	y	2 X W SOFT	Y	2 X W SOFT
6	y	4 X SOFT	Y	4 X SOFT	Y	4 X SOFT	Y	4 X SOFT	Y	4 X SOFT	Y	4 X SOFT
7	y	2 X W SOFT	y	2 X W SOFT	y	2 X W SOFT	y	2 X W SOFT	y	2 X W SOFT	y	2 X W SOFT
8	y	4 X SOFT	y	2 X W SOFT	y	2 X W SOFT	y	2 X W SOFT	y	2 X W SOFT	y	2 X W SOFT
9	y	2 X W SOFT	y	2 X W SOFT	y	2 X W SOFT	y	2 X W SOFT	y	2 X W SOFT	Y	1 X W SOFT
0	y	2x w soft	y	2x w soft	y	2x w soft	y	2x w soft	y	2x w soft	y	2 X W SOFT
11	y	2x w soft w mittens	y	2x w soft w mittens	y	2x w soft w mittens	y	2x w soft w mittens	y	2x w soft w mittens	y	2 X SOFT W/MITTS
12	y	2x w soft	y	2x w soft	y	2x w soft	y	2x w soft	y	2x w soft	y	2x w soft
13	y	4 X PINEL W/MITTS	y	4 X PINEL W/MITTS	y	4 X PINEL W/MITTS	y	4 X PINEL W/MITTS	y	4 X PINEL W/MITTS	y	4 X PINEL W/MITTS
14	y	2x w soft	n		y	1x w soft (other in brace)	y	1x w soft (other in brace)	y	1x w soft (other in brace)	y	1x w soft (other in brace)
15	n		n		n		n		n		n	
16	y	2x w soft	y	2x w soft	y	2x w soft	y	2x w soft	y	2x w soft	y	2 X W SOFT
17	y	4x soft @12:30 4 X PINEL	y	PINEL X 4	y	PINEL X 4	y	PINEL X 4	y	PINEL X 4	y	PINEL X 4
18	N		N		N		N		Y(FOR ENTUBE INSERTION)	2 x soft w	Y	2 X W SOFT
19	Y	SOFT X 2 W	Y	2 X W SOFT	Y	2 X W SOFT	Y	2 X W SOFT	Y	2 X W SOFT	Y	2 X W SOFT
20	Y	2 X SOFT W	Y	2 X W SOFT	Y	2 X W SOFT	Y	2 X W SOFT	N (REMOVED TO DEAL W/ PIV		Y	SOFT X 4
21	Y	2 X SOFT W	Y	2 X SOFT W	N		Y	2 X SOFT W	Y	4 X SOFT	Y	SOFT X 4
22	N		N		N		N		N		N	
23	Y	4 X SOFT	Y	4 X SOFT	Y	4 X SOFT	N		Y	4 X SOFT	Y	4 X SOFT
24	Y	4 X SOFT W/ MITTS	Y	4 X SOFT	Y	4 X SOFT	Y	4 X SOFT W/MITTS	Y	4 X SOFT	Y	4 X SOFT
25	Y	4 X SOFT W/MITTS	Y	4 X SOFT W/MITTS	Y	4 X SOFT W/MITTS	Y	4 X SOFT W/MITTS	Y	4 X SOFT W/MITTS	Y	4 X SOFT W/MITTS
26	Y	2 X W SOFT	Y	2 X W SOFT	Y	2 X W SOFT	Y	2 X W SOFT	Y	2 X W SOFT	Y	2 X W SOFT
27	N		N		N		N		N		N	
28	y	2x w soft + mitts	y	2x w soft + mitts	y	2x w soft + mitts	y	2x w soft + mits	y	2x w soft + mits	Y	2 X W SOFT W/MITTS
29	N		N		N		N		N		N	

30	Y	2 X W SOFT	Y	2 X W SOFT	Y	2 X W SOFT	Y	2 X W SOFT	Y	2 X W SOFT	Y	2 X W SOFT
31	Y	4 X SOFT	Y	4 X SOFT	Y	4 X SOFT	Y	4 X SOFT	Y	4 X SOFT	Y	4 X SOFT
32	Y	2 X SOFT	Y	2 X SOFT	Y	2 X SOFT	Y	2 X SOFT	Y	2 X SOFT	Y	2 X SOFT
33	Y	4 X SOFT	Y	4 X SOFT	Y	4 X SOFT	Y	4 X SOFT	Y	4 X SOFT	Y	4 X SOFT
34	Y	2 X W SOFT	Y	2 X W SOFT	Y	2 X W SOFT	N		Y	2 X W SOFT	Y	2 X W SOFT
35	N		N		N		N		N		N	

Supplementary Table 2: Use of physical restraints Y/N – Intervention Arm

Participant	Hour 0	Type	Hour 1	Type	Hour 2	Type	Hour 3	Type	Hour 4	Type	Hour 5	Type
1	y	2 X PINEL W	y	2 X PINEL W	y	2 X PINEL W	y	2 X PINEL W	y	2 X PINEL W	y	2 X PINEL W
2	Y	4 X SOFT	Y	4 X SOFT	Y	2 X W SOFT	Y	2 X W SOFT	Y	2 X W SOFT	N	N/A
3	y	2 X W SOFT	y	2 X W SOFT	y	2 X W SOFT	y	2 X W SOFT	y	2 X W SOFT	y	2 X W SOFT
4	y	2 X SOFT	y	2 X SOFT	n				n		n	
5	y	pinels x4	y	pinels x 4	y	pinels x4	y	pinels x4	y	pinels x4	y	PINEL X 4
6	y	2 X W SOFT	y	2 X W SOFT	y	2 X W SOFT	y	2 X W SOFT	y	2 X W SOFT	y	2 X W SOFT
7	n		n		n		n		n		n	
8	y	4 X SOFT W/MITTS	y	4 X SOFT W/MITTS	y	4 X SOFT W/MITTS	y	4 X SOFT W/MITTS	y	4 X SOFT W/MITTS	y	4 X SOFT W/MITTS
9	N		n		n		n		n		n	
10	Y	2 X W SOFT W/MITTS	y	2 X W SOFT W/MITTS	y	2 X W SOFT W/MITTS	y	2 X W SOFT W/MITTS	y	2 X W SOFT W/MITTS	y	4 X W SOFT W/MITTS
11	y	pinels 4x + waist belt	y	4x pinel	y	4x pinel	y	4x pinel	y	4x pinel	y	4 X PINEL
12	N		N		N		Y	Posey Chair	N		Y	2 X w soft
13	n	1:1 SITTER	n	1:1 SITTER	n	1:1 SITTER	n	1:1 SITTER	n	1:1 SITTER	n	1:1 SITTER
14	n		n		n		n		n		n	
15	n		n		n		n		n		n	
16	y	2x soft	y	2x soft	y	2x soft	y	2x soft	y	2x soft	y	2 x w soft
17	y	4x soft	y	4x soft + sitter	y	4x soft	y	4x soft	y	4x soft	y	4x soft
18	y	2x w soft	y	2x w soft	y	2x soft w	y	2 X W SOFT	y	2x soft w	y	2 X W SOFT
19	Y	2X W SOFT	N		N		Y	2X W SOFT	Y	2x w soft + mits	Y	2X W SOFT
20	Y	4x soft	Y	4x soft + sitter	Y	2 X SOFT	Y	2 X W SOFT	Y	2 X SOFT	Y	2 X SOFT
21	Y	2 X SOFT W	Y	2 X SOFT W	Y	2 X SOFT W	Y	2 X SOFT W	Y	2 X SOFT W	Y	2 X SOFT W
22	Y	4 X SOFT W/ MITTS,	Y	4 X SOFT W/ 1 MITT	Y	4 X SOFT W 2 MITTS	Y	4 X SOFT W/ MITTS	Y	2X SOFT W W/MITTS	Y	2 X SOFT W W/MITTS
23	Y	2 X W SOFT	Y	2 X W SOFT	Y	2 X W SOFT	Y	2 X W SOFT	Y	2 X W SOFT	Y	2 X W SOFT
24	N		N		N		N		N		N	
25	Y	2 X W SOFT	Y	2 X W SOFT	Y	2 X W SOFT	Y	2 X W SOFT	Y	2 X W SOFT		
26	Y	4 X SOFT	Y	4 X SOFT	Y	4 X SOFT	Y	4 X SOFT	Y	4 X SOFT		
27	Y	R X 4 SOFT	Y	4 X SOFT	Y	4 X SOFT	Y	4 X SOFT	Y	4 X SOFT		
28	Y	SOFTX 1W R	Y	1 X SOFT W	Y	1 X SOFT W	Y	1 X SOFT W	Y	1 X SOFT W		
29	Y	2 X SOFT W	Y	2 X SOFT W	Y	2 X SOFT W	Y	2 X SOFT W	Y	2 X SOFT W		
30	N		N		N		N		N			
31	Y	2 X SOFT W	Y	2 X SOFT W	Y	2 X SOFT W	Y	2 X SOFT W	Y	2 X SOFT W		

32	Y	2 X SOFT W	Y	2 X SOFT W	Y	2 X SOFT W	Y	2 X SOFT W	Y	2 X SOFT W		
33	Y	2 X SOFT W	Y	2 X SOFT W	Y	2 X SOFT W	Y	2 X SOFT W	Y	2 X SOFT W		
34	Y	4 X SOFT	Y	4 X SOFT	Y	4 X SOFT	Y	4 X SOFT				
35	N		N		N		N					

W= Wrists, Soft denotes soft restraints. Where 4 x are noted this indicates wrist and ankle restraints. Pinel restraints are considered hard restraints. Mitts= mitten like hand coverings placed over patients hands to stop them having the ability to use their fingers to untie restraints, pull out lines or scratch themselves or healthcare workers.

Supplementary Note 2: Study Protocol

Use of a novel interactive digital therapeutic intervention for the management of delirium in the critical care environment

Study Protocol

Version 1.6

Oct 27th 2021

Principal Investigator
Name: Steven Reynolds
Title: Dr
Clinical Program: Critical Care
Department: Intensive Care Unit, Royal Columbian Hospital

Co-Investigator
Name: Michelle Nicholas
Title: RN
Program: Critical Care
Department: CSICU, Royal Columbian Hospital

Co-Investigator
Name: Suzette Willems
Title: RN- Research Coordinator
Program: Critical Care
Department: Critical Care, Royal Columbian Hospital

Research Team Member
Name: Mangatpreet Vohra
Title: Patient Partner

TABLE OF CONTENTS:

Introduction and Background	Page 3-7
Purpose and Justification	Page 7-8
Research Question	Page 8
Research Objectives	Page 8
Research Hypothesis	Page 8
Study Design	Page 9-10
Generalizability of findings	Page 10
Recruitment	Page 11
Inclusion Criteria	Page 11
Exclusion Criteria	Page 11
Enrollment of Participants	Page 11-12
Consent	Page 12-14
Randomization	Page 14
Intervention	Page 14-15
Standard Care	Page 15
Study Visits	Page 15
Follow up Visits	Page 15-16
Data Collection	Page 16
Outcome Measures	Page 17-18
Sample Size	Page 19
Statistical Plan	Page 18
Subject Safety Provisions	Page 18-20
Serious Adverse Event Reporting	Page 20-21
Ethical Considerations: Risks and Benefits	Page 21-22
Ethics Approval	Page 22
Plans for Publication	Page 22
References	Page 23-26

INTRODUCTION AND BACKGROUND

Introduction:

Delirium is a fluctuating state of confusion and agitation that affects between 30-60% of acute care patients annually and as many as 80% of critical care patients[1]. Delirium is rapid in its onset and may persist for as little as hours or as long as multiple weeks. It is categorized in to three types; hypoactive, hyperactive and mixed where patients can fluctuate between states. Hyperactive delirium, while less prevalent attracts significant clinical attention and resources due to associated psychomotor agitation which complicates care. Patients may experience hallucinations or delusions and become aggressive or combative posing a risk of physical harm to themselves and healthcare staff.[2,3] Delirium has wide reaching implications in terms of financial cost to the healthcare system. It has been quoted as costing the US healthcare system in the region of 38-152 billion dollars annually in extended hospital stays, resource allocation and reliance on pharmacological therapies.[4,5] Delirium complicates the provision of care by healthcare staff, has been linked to increased risk of death and poor overall outcomes.[1,2,6] Some of the difficulty in identifying effective strategies for delirium is the multitude of precipitating or contributing factors that may lead to its development. Those with underlying brain health issues such as dementia are already at a pre-disposed risk of the condition developing. Imbalances in electrolytes, polypharmacy, sleep disturbance, underlying disease process and or surgical intervention, all commonplace in the critical care population are considered risk factors. Due to its multifactorial nature, it is important to examine and correct the underlying disease etiology wherever possible. Standard care is still largely reliant on chemical and physical restraints. Pharmacological agents can carry significant risk and side effects and there is little proof that any singular non-pharmacological intervention is successful in mitigating delirium.[6–8] A multi-modal approach to management that encompasses not just delirium but also pain, agitation, immobility and sleep disturbance is now recommended in the current PADIS (Pain agitation delirium immobility and sleep disruption) clinical guidelines for Intensive Care Unit (ICU) patients and the ICU Liberation Bundle.[6,9] This includes the maintenance of sleep cycles, early mobilization, frequent reorientation and measures to

reduce pain, anxiety and agitation. Within Fraser Health, the focus on strategies to prevent and reduce delirium is considered a high value issue. The introduction of taskforces and working groups aimed at this subject highlight the health authorities' commitment to finding realistic working solutions to this issue.

Recommendations for management, using non-pharmacological interventions are made on the basis of generally poor-quality studies. The 2018 PADIS guidelines[6] rate the majority of recommendations as having low or very low quality of evidence. As clinicians, we are still searching for effective strategies to ensure the best possible outcomes for our patients. The Royal Columbian Hospital in partnership with Mindful Garden Digital Health Inc is proposing a series of iterative clinical trials of a novel digital interactive behavior modification platform. The display produces nature imagery, a "virtual garden" in response to patient movement and vocalization and aims to reduce anxiety and psychomotor agitation in the hyperactive delirious critical care population. The initial trial will determine the potential effectiveness of using this platform in the critical care patient populations to reduce reliance on unscheduled medication administration. It will also provide the basis of a framework for a series of iterative studies where one aspect of the device is altered at a time and is planned to include variations in visual content, incorporation of sound output, further bio-feedback mechanisms with wearable sensors, dose dependent responses and a broader range of target populations. The initial study is predicted to be conducted over a period of 4-6 months in the ICU and HAU at the Royal Columbian Hospital in New Westminster with subsequent iterative studies taking 3-4 months with a planned 6-8 total over a course of approximately 2 years.

Background:

Delirious patients may experience visual or auditory hallucinations, disorganized thinking or inattention as well as difficulty problem solving or conducting simple tasks. Risks of physical injury to both patients and staff from compulsive or combative patients often results in the use of both physical and chemical restraints.[10–12] While least restraint policies may be in place in a number of institutions, practicalities and logistics of care often see them being employed in the intensive care environment despite potential negative physical and psychological effects.[10] The need to ensure the safety of patients with endotracheal tubes, large intravenous lines, invasive therapies and monitoring is often cited as reasoning for the employment of physical restraints despite limited evidence to support their efficacy.[6,13] Rose et al [10] indicated as high as 76% use in intensive care units and Luk et al[12] reported 50 % use in Canadian ICU's. Some evidence in fact points to higher rates of adverse events such as self-extubation when restraints are in place. [14,15] Despite apparent widespread use, the 2018 PADIS guidelines declined to provide any clear recommendation for or against physical restraints.[6]

Where physical restraints are not sufficient or employed, chemical restraint with the use of anti-psychotics and sedatives are commonplace. These medications are used more as a method to control agitation and is often seen as the safe option among nursing staff who are frustrated with trying to manage agitated patients.[2] Sedating and physically restraining intensive care patients can be emotionally distressing for families and caregivers at a time that is already fraught with difficulties.[16]

Pharmacological interventions are widely used despite limited proof of their utility in reducing the severity or length of delirium.[17–19] They are routinely administered on a PRN (pro re nata), or as needed basis by nursing staff as a method to manage symptoms of agitation, despite current guidelines recommending sparing usage as an exception rather than a primary management strategy.[6,20] Extra-pyramidal effects, risk of QT interval prolongation on electrocardiogram (ECG) and other toxicities exist with many of the current medications.[21,22]. Medications are routinely prescribed as nighttime sedation aiming to maintain day night routines and adherence to natural circadian rhythms. All of these medications carry risk and the 2018 PADIS guidelines warn against over sedating patients, recommending daily breaks from sedation and light sedation as a goal of care.[6] Current practice in the RCH ICU reflects this with daily sedation vacations being routinely incorporated in to care wherever deemed clinically safe for the patient. More recently, novel alpha 2 agonists such as dexmedetomidine have shown utility as a sedation agent to allow weaning from mechanical ventilation in agitated and delirious populations. It has also shown a reduced reliance on opioids.[23] Atypical sedatives and antipsychotics like dexmedetomidine, have gained favor amongst clinicians as effective aids to wean from mechanical ventilation, but can have negative cardiac effects and carry significant financial cost compared with more traditional sedation agents.[24]

Multiple non-pharmacological interventions have been trialed. Music therapy has shown some success in reducing symptoms of anxiety and agitation as seen in the study by Cooke et al (2010).[25] This study focused on dementia populations and should be viewed with some caution when generalizing to acute care populations. Due to the nature of ICU environments this may not always be a practical intervention but was successfully implemented in a study by Johnson et al[26] in a trauma ICU. Participants in this study were delirium positive on admission but both study arms

measured negative throughout the course of the trial and the authors did not include patients who were mechanically ventilated. Despite the issues surrounding these trials music therapy is recommended in the PADIS guidelines.[6] An auditory component is planned for inclusion in the proposed iterative studies of the Mindful Garden (MG) intervention.

A focus on frequent re-orientation of delirious individuals to time and place, as well as maintenance of the day night routine and circadian rhythms are widely used non-pharmacological approaches to patient management in the literature as well as in practice within Fraser Health. Ensuring eyeglasses and hearing aids are utilized to optimize sensory input, as well as informational boards within the patient's visual field with details about their environment help in reinforcing a basis in reality. These are routinely incorporated in to care within RCH's critical care units and can be considered very low risk interventions. A systematic review conducted by Bannon et al (2019)[27] showed no benefit in pooled analysis of any of the non-pharmacological interventions tested in reducing the incidence or duration of delirium. The review also highlighted the low quality of evidence produced by these studies citing indirectness of effect and variable or unclear outcome measures.

New directions in interventions have been emerging out of the technology sector related to computer gaming. Virtual reality (VR) and the use of immersive digital environments have largely been tested in pediatric and burn populations as a means of providing distraction during painful procedures.[28–31] Turon et al[32] conducted a pilot study in an ICU where an interactive screen was used to provide neurocognitive training sessions aimed at improving quality indicators and long term neurocognitive outcomes. This system was not studied in confused or delirious populations and required awake alert participants to actively complete tasks in training sessions. Use of vital signs such as heart rate variability and mean arterial pressure indicated the intervention was safe in terms of physiological effect but no cognitive outcomes were reported. Gerber et al (2017) conducted a pilot study in mock ICU setup where healthy volunteers were tested for safety and feasibility of using VR headsets and noise cancelling headphones to deliver nature imagery and music or nature recordings. The authors believe that delivery of nature imagery stimulates a parasympathetic response promoting a positive emotional state as well as allowing attentional capacity to be restored as supported by both stress recovery and attention restoration theory.[33–35] Gold and Mahrer [36] further suggest that the emotional connection experienced during computer games, and in turn interactive VR headsets activate the amygdala and hippocampus to create memory, leading to a positive connection and is more likely to provide positive outcomes. There are significant considerations to be made when assessing the feasibility of such therapies within the hyperactive delirious critical care population. Headsets or headphones of VR equipment may in themselves cause agitation or heightened anxiety in those suffering with altered cognition. Loss of perception of the surrounding environment, discomfort of the equipment or feelings of claustrophobia amongst some patients is possible. Patients in critical care often have significant amounts of equipment already attached making it difficult to then place more on the patient especially if bed bound, or with injuries and dressings. Patient positioning must also be considered when using this type of equipment as side positioning, important for reduction of pressure areas and possibly a requirement with certain injuries, is likely unattainable during periods of equipment use. Placing a VR headset on a patient in an anxious state may be possible, but with significantly restless or agitated patients keeping a headset in place is unlikely to be achievable.

Mindful Garden is a novel, patient-responsive digital behavioural modification platform. It has been developed to target the anxiety and agitation associated with hyperactive delirium. The platform utilizes a mobile, high-resolution screen-based digital display with sensor technology. MG layers 2D video of real nature imagery with hyper-real 3D animations in direct response to patient agitation and restlessness for which movement and vocalization are considered the initial surrogate markers. A built-in camera system and microphone use proprietary algorithms to compare approximately 140 measures per second for both movement and vocalization input to determine fluctuations in these markers and drive on screen content delivery. Hyper-real animations of growing and receding flowers in addition to butterflies in flight are produced in a volume that is directly responsive to measured patient behaviour (See Fig 1.0)

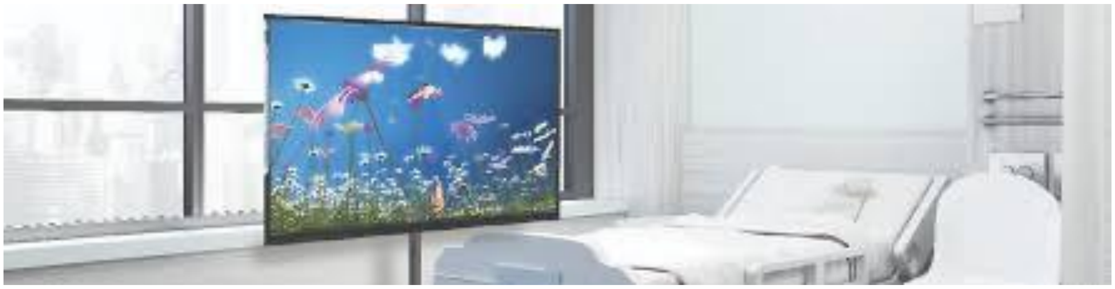

Fig 1.0

As the patient calms and measurable agitation reduces, the volume of hyper-real images on screen reduces. MG is likened to a computer game that the patient is unaware they are participating in. Utilization of a digital screen with nature imagery may provide neuro-cognitive and psycho-physiological benefits such as those described in studies using Virtual Reality (VR), immersive digital environments and exposure to natural environments without some of the practical challenges. Incorporation of an interactive component to the dynamic visual content may be effective as a de-escalation tool for psychomotor agitation. MG is mobile, requires no physical attachment to the patient, is implemented with minimal effort and training to healthcare staff, and does not require active management or observation by staff when in use with patients. Initial unpublished validation data from use of MG in a long-term care dementia population (non-FHA Study) showed a 46% positive rating from staff in successful agitation reduction when MG was used as a behavioural “crash cart” for management of self manifesting agitated behaviours with a 0% negative rating (13 exposure events for 7 participants) (Principal Investigator Dr Gloria Gutman).[37] Mindful Garden has been successful in gaining recognition through grants from AGE-WELL and Center for Aging and Brain Health Innovation. This has enabled expanded product testing internationally and consideration of alternate target populations. This would be the first trial of this product in an acute care environment, allowing Fraser Health to be at the forefront of this as an accepted non-pharmacological treatment intervention.

Purpose and justification

There is a human and financial cost to delirium. Delirium is a substantial issue not just within Fraser Health but globally. To date, the majority of available interventions have shown limited proof of success. In early 2020 Fraser Health named delirium as one of its 5 patient safety priority areas for the 2020-2022 period and has committed considerable time and effort to finding viable working solutions to prevent and manage delirium within its patient population. By conducting this study, the research team aims to add to the toolbox of care available to healthcare practitioners. In using a novel digital therapeutic approach, we hope to overcome some of the side effects, risks and potential toxicities related to standard treatment. This may ultimately result in improved outcomes for patients as well as a safer work environment for healthcare staff and an improved experience for family members and this vulnerable patient population. This study may also place Fraser health and the Royal Columbian Hospital at the forefront of a new therapeutic option in a healthcare field that is rapidly expanding in a global context.

Research Question:

Does use of the Mindful Garden digital behavior modification platform reduce or normalize the level of measurable agitation in adult acute care population compared to standard care alone?

The studies primary outcome aim is to compare agitation scores in adult hyperactive delirious critical care patients in 2 groups, those exposed to the Mindful Garden digital behavior modification platform, and those receiving standard care alone.

Research Objective(s):

Primary Objective:

In conducting this randomized clinical trial, we aim to answer whether the use of MG in addition to standard care in critical care patients, with mixed or hyperactive delirium, results in a reduced agitation scores RASS-(Richmond agitation and sedation scale).[38]

Secondary Objectives:

- To compare the difference in the following secondary outcome measures between those receiving the intervention and those receiving standard care alone
    i. Use of physical restraints, whose use is associated with negative outcomes.
    ii. Agitation and delirium scores ICDSC (Intensive care delirium screening checklist)[39]
    iii. Unplanned removal of devices such as naso-gastric tubes, venous and arterial lines and endotracheal tubes
- To examine the trends in measured activity throughout the intervention period for those in the intervention arm (Movement count average and measured sound input)
- To examine the effect on and trend in physiological parameters over the study period including heart rate (HR), heart rate variability (HRV), respiratory rate (RR) oxygen saturation (Sa02) and mean arterial blood pressure (MAP) as well as the use of vasopressors commonly used for the management of hemodynamics in the critical care environment
- Determine the treatment effect and sample size for planned iterative trials
- To examine user evaluation metrics through the use of surveys to caregivers and family members
- To answer whether the use of MG in addition to standard care in critical care patients, with hyperactive delirium, results in a reduced reliance on unscheduled anti-psychotic or sedative medications, which have significant toxicities.

Research Hypothesis(es):

Use of the Mindful Garden behavioral modification platform will result in reduced or normalized agitation scores in the adult delirious acute care population compared to standard care alone.

Methods:

Study Design:

The initial study will be a single center, open label randomized interventional clinical trial. It will be conducted at an acute care facility (Royal Columbian Hospital-RCH) enrolling patients in both the Intensive Care Unit and High Acuity Unit of the hospital. An iterative trial design will then be followed for a total of 6-8 phases adjusting one variable of the MG design or treatment delivery in each successive iteration.

Trials 2-8:

The initial trial will provide an accurate estimation of the effect size and sample size required for subsequent trial cycles in addition to evaluating the efficacy of the base intervention in achieving the stated outcomes. An iterative trial design will then be followed for a series of 3-4 month experiments, with a planned total of 6-8 cycles. The sample size will be smaller because all recruited patients will be entered into the experimental arm and compared to the initial control group. Although this is not a true randomized trial for the subsequent studies, it facilitates rapid and nimble assessments of multiple variables in a relatively short time frame by altering one at a time. These trials will be similar in design to the first experiment but change a single variable. At the end of each experiment data analysis will allow the research team to determine go/no-go decisions regarding incorporation of the studied variable into subsequent testing cycles on the basis of enhancement, detraction or neutrality in achieving the study aims in comparison to the base or previous intervention variable. This stepwise approach will lead to the most efficacious version of the intervention with rigorous clinical validation. If it becomes evident that the baseline intervention is not effective in the target population of critically ill delirious patients, early advancement to alternate target populations will be made such as peri-operative use in elderly cardiac and orthopedic surgery patients who are at very high risk of developing delirium.[40,41]

During trials 2-8 it is intended that the following adjustments may be made. Where required amendments of the protocol will be submitted for review by the REB in advance of the specific trial commencement. As each iteration will be determined in part by the results of the previously completed studies, this list may not be completely accurate to the order and nature of each iterative study.

Planned iterations**:**

- Inclusion of sound output.
  - It is thought that emitting sound at the 528Hz frequency may enhance the calming and anxiety reducing effect of the visual platform. A sound selection at this frequency is yet to be determined.
  - Inclusion of sound output: white noise. It is possible that white noise emission may be calming and help to mask or cancel out the surrounding noises of the patient care environment. This may show benefit in the outcomes being measured compared to the base platform.
- Alternate visual content
  - Alternate dynamic visual output may be compared. This may be one alternate version such as a water or beach scene that may be tested.
  - A choice of patient/substitute decision maker selected visual content may be tested to determine if providing a choice of the visual content may be more impactful on the outcomes being measured.
- Use of eye tracking
  - Eye tracking technology may be incorporated to drive the delivery of on-screen content and to determine the level of interactivity with the platform by participants
- Use of bio-sensors to drive on screen content
  - Use of bio-feedback sensors may be incorporated to drive on-screen content using metrics such as heart rate, heart rate variability, respiratory rate. As this would potentially require attachment of a sensor to the participant, a change in device classification may be needed and an amendment of the ethics certificate will be applied for once determinations for this iteration is made. If it is

determined that a device classification change is required, an interim testing authorization (ITA) will be requested from Health Canada.

- Peri-operative care patient populations

    - Advancement into this patient population may be made using an adjusted approach of the protocol. Determinations for this will be made after completing initial iterations and any deviations to the current protocol will be applied for as amendments to the REB.

Generalizability of findings:

Due to the overwhelming prevalence of delirium in the acute care population, there is significant potential benefit to be gained by patient populations within and extending beyond critical care. Results may be generalizable outside of the critical care setting for patients throughout acute care as well as those suffering with more chronic mental health disorders of altered cognition where anxiety and agitation are key components of the condition.

Sampling Design and Subject Selection:

Recruitment:

Recruitment is planned to achieve 3-5 participants per week taking 14-23 weeks for the initial trial, with subsequent cycles expected to take 3-4 months. RCH has 30 ICU/HAU beds that routinely operate at maximum capacity. An estimated 6-admissions per day across units (20% bed turnover rate) equates to 30 admissions Monday to Friday, placing recruitment at 10-16%. MG Digital Health Inc will provide 2-4 physical devices enabling same day enrollment of multiple participants. The burden to nursing staff will be minimized and within scope with staffing ratios of 1:1 or 1:2 dependent on patient acuity and unit. Participants recruited outside of critical care may have study measurements conducted by research personnel to limit impact on nursing workload and workflow. Psychiatry clinical liaison teams receive between 3-5 new referrals daily for the management of delirium at RCH which will further aid in reaching planned recruitment numbers.
Recruitment will be conducted by the research coordinator or trainee in accordance with ethics and regulatory requirements.

Inclusion Criteria**:**
- Adult (age >18yrs)
- Admitted to RCH
- RASS +1 or greater for 2 assessments at least 1 hour apart within the 24-hour period directly prior to study enrollment and persisting at the time of enrollment or equivalent documentation of agitation related to delirium for participants admitted outside of critical care
- Demonstrated incidence of at least 2 PRN medication events for the management of delirium-associated agitation in the preceding 24 hours/and or infusion of psycho-active medication for the management of delirium (eg: dexmeditomodine)
- ICDSC greater than or equal to 4 at time of enrollment or CAM (Confusion assessment method) positive screening for those admitted outside of critical care. [42]

Exclusion Criteria**:**
- Planned procedure or test that precludes participation in the full 4-hour study session
- Unable to see (visual impairment such as documented blindness, ongoing inability to keep eyes open or documented or assessed inability to focus, track or maintain visual contact for extended periods as determined by recruitment personnel)
- Significant uncontrolled pain with a Verbal Pain scale of 5/10 or greater or a Clinical Pain Observation Tool >4 despite intervention at the time of enrollment.
- RASS of 0 to -5 at study enrollment indicating patient is awake and calm or sedated
- Refusal for participation by the participant's responsible physician

- Currently enrolled in any other research study involving drugs or devices which could impact on the outcomes of interest, as evaluated by the Principal Investigator

Method of Recruitment:

Enrollment of participants:

Participants will be identified by the clinical research coordinator or PhD trainee daily in collaboration with critical care department medical and nursing staff. Where potential participants are admitted outside of critical care, identification of participants and referral to the research coordinators will made by the psychiatric clinical liaison team and/or in collaboration with local nursing staff.
Baseline data regarding patient demographics and underlying disease conditions will be collected by study personnel at time of enrollment and screened for inclusion/exclusion criteria and entered in to standardized clinical reporting forms (CRF). All efforts will be made to initiate the protocol after daily multi-disciplinary rounds. Major changes in the direction of care for participant's delirium management, specifically non-urgent orders will be delayed until after the study period. Urgency will be determined by the participants care team. Patients will not be eligible for multiple enrollments to avoid biasing of a training or repeated exposure effect.

Informed Consent:

Waived consent is being applied for and will be carried out with processes in accordance with local regulatory ethics board and privacy department requirements. Where possible after the fact, informational letters will be provided to participants substitute decision makers (SDM) and can be explained by the clinical research coordinator or trainee as requested. Participants and their SDM's will have to option to remove the participant from inclusion in the trial. As the participants have a disorder of altered cognition, they are not able to give informed consent.

Waived consent is being requested in accordance with the TCPS guidelines Article 3.7A ensuring the following conditions are met:

a) **the research involves no more than minimal risk to the participants:**

   As MG is a non-invasive digital platform that has no physical attachment to the participant it is considered a Class I medical device in accordance with Health Canada device classifications Rule 12 https://laws-lois.justice.gc.ca/eng/regulations/SOR-98-282/page-12.html#h-1022100. This considers it of low risk and does not require an Investigational Testing Authorization to conduct the trial. Patients often receive exposure to digital media, use of TV's, music and distraction techniques as part of the standard course of care. The investigators consider this intervention to be of similar risk as these interventions and within the limits of what a participant would be exposed to in the course of a normal ICU stay. Although there is an interactive component to the intervention, wherein a camera and microphone measure activity to drive onscreen content, there is no recording capability of this equipment and no active participation of the patient is required.

b) **the alteration to consent requirements is unlikely to adversely affect the welfare of participants:**

   The intervention being tested is non-invasive and the observational measures being recorded are routinely conducted as a matter of standard care in the critical care environment. As this intervention is being used as an adjunct to standard care, there is no loss of the normal course of care that might adversely affect the participant or place them at risk. The majority of data will be collected from the participants medical records and as such waived consent will not disadvantage or adversely affect participants. Participants or their substitute decision makers will be given the opportunity to withdraw their information from inclusion in the study.

c) **it is impossible or impracticable (see Glossary) to carry out the research and to address the research question properly, given the research design, if the prior consent of participants is required:**

Delirium is a waxing and waning condition of altered cognition. This places strain on the recruitment process in capturing participants in a hyperactive phase of their delirium for inclusion. As this makes enrollment and the study start time sensitive it is not feasible to attempt to conduct a full informed consenting process prior to inclusion. Participants are unable to provide consent themselves, due to the presence of altered cognition and therefore consent would need to be sought from the participants substitute decision maker. This may add significant time delays and complexities. Additional restrictions on visiting and presence of family members due to the current pandemic would require consent to be conducted over the phone which may cause additional delays due to difficulty in reaching SDM's. Additionally, due to privacy regulations contact for purposes outside of a clinical context are restricted and so gaining consent prior to the commencement of the study is impracticable. It may realistically take some hours, or days before face to face contact with a participants SDM is possible.

d) **in the case of a proposed alteration, the precise nature and extent of any proposed alteration is defined**

Waived consent is requested for the initial trial of the intervention of 70 participants. Waived consent is also requested for subsequent iterations where there is not any significant changes to the nature of the interventions invasiveness. If iterations progress to having any physical attachment to the patient, or MG is changed such that an associated change in device classification is required then the consent process will be evaluated and amended in accordance with REB requirements.

e) **the plan to provide a debriefing (if any) that may also offer participants the possibility of refusing consent and/or withdrawing data and/or human biological materials, shall be in accordance with Article 3.7B.**

Where participants regain capacity after inclusion a letter of information will be provided to them outlining the details of their inclusion in the study and providing details to allow them to withdraw their information from the study. If SDM's are present within the hospital or contacted within the course of care in a clinical context, consent for contact by the research coordinator will be requested by the clinical staff. If this is obtained, the research coordinator will make contact with the SDM and request permission to provide the informational letter which outlines the inclusion in the study and gives information about the right to withdraw participants data from the study. If participants or their SDM's agree to further contact, then a follow up phone call at 30 days may be made to ensure continued agreement to keep the participants data in the study or give them the option to withdraw.

Study Procedures:

Randomization:

Randomized, 1:1 open label, interventional clinical trial using random probability sampling. Patients will be assigned a sequential enrollment number from 001-070 and matched to trial condition (intervention or control) using an online randomization tool created and maintained by CHEOS (Centre for Health Evaluation and Outcome Sciences). Alternately a randomization list as supplied by CHEOS will be sealed in to sequentially numbered envelopes (numbered according to sequential enrolment) by a designated non-study member. If using sealed envelopes, on enrollment the clinical research coordinator or research assistant will access and open the sequentially sealed envelope and assign the trial condition according to contents (control/intervention). Sealed envelopes will be stored in a locked cupboard on hospital property in accordance with local privacy regulations. CHEOS will retain the master randomization list however as this is an open label trial it is not expected that breaking of the randomization code and master list will be required in any event.

Intervention**:**

Experimental arm:

For those randomized to the experimental arm the MG digital display will be placed near or at the foot end of the bed or in sight of the participant should they be in a chair at the time of the study. The screen will be placed such that visualization by the participant is possible, but out of physical reach of the patient to ensure that damage or harm to the patient or damage of the equipment is not possible from grabbing or kicking the device unit by participants. As the MG display is a mobile device on a wheeled frame with an articulating arm the position can be adjusted to ensure it is maintained within the participants field of vision at all times. The frame has locking wheels and runs on an inbuilt battery to minimize trip hazards and reduce the need for repositioning of equipment to allow access to power points. A retractable power cord is attached as a backup should the battery not be functional for any reason or not have the required power to last for the full intervention period. The device will be set up and initialized by the research coordinator or research assistants according to operating manual guidelines. For the provision of care such as mobilization, bathing or turning that requires physical interaction with the participant, the interactive component (voice and mobilization input from the camera and microphone system) of the device can be placed in standby mode by nursing staff or research coordinator for 5-minute intervals. An inbuilt timer will automatically restart the device at the 5-minute mark. If this is not enough time to complete care, the standby feature can be activated again as required. As the device is responsive to changes in physical activity and vocalization this will ensure that delivery of on-screen animations will be in response to measured participant activity related to agitation and not to activity of additional persons moving within the patient care area as much as possible. For patients who are mechanically ventilated the microphone function will be disabled to avoid auditory activation by the ventilator. The research coordinator or research assistant will record and monitor the time of intervention delivery which is not to exceed 4 hours total inclusive of periods of standby to directly compare to control participants. Early termination of the intervention due to non-tolerance of the intervention (as determined by the participants responsible physician or care team) will be analyzed per protocol according to intention to treat analysis. Paper based surveys of caregivers and family members will be conducted where possible at the end of the intervention period using 9-point Likert scales and collected by study coordinator or research assistant. Mindful Garden study units use input from the mounted camera to measure movement and sound generation as surrogate markers of agitation. This input drives the on-screen content delivery using proprietary algorithms in direct response to the level of measured agitation. This measurement generates activity logs within the hard drive of the unit represented numerically in tabular form (see table 1). Measures are generated approximately every 2 secs throughout the active study period. Each session has a unique identifier and is also recognizable through date and time stamps. No patient identifying data is included, only the unique participant enrolment number that is entered in-to the system at the commencement of the study session.

1: MOVEMENT: This is calculated by comparing the difference in pixel density from the previous frame to the current one. The resulting value is then averaged over the collected frames and returned as a decimal percentage of change, called the Movement Count Average. Values are between 0 and 1 with 0 showing the lowest amount of activity and one the highest

2: VOLUME: This is calculated by finding the highest level of sound, converted to a decimal percentage between 0 and 1 (0 being the lowest level and 1 the highest)

Table 1: example of activity logs

_id	sessionId	sensorType	sensorValue	phase	createdAt
60427b2077f32877e8fdb04f	60427b1e77f32877e8fdb04e	MOVEMENT	0.024789	EXPERIENCE	Fri Mar 05 2021 10:40:32 GMT-0800 (Pacific Standard Time)
60427b2077f32877e8fdb050	60427b1e77f32877e8fdb04e	VOLUME	0.000702	EXPERIENCE	Fri Mar 05 2021 10:40:32 GMT-0800 (Pacific Standard Time)
60427b2277f32877e8fdb051	60427b1e77f32877e8fdb04e	VOLUME	0.000349	EXPERIENCE	Fri Mar 05 2021 10:40:34 GMT-0800 (Pacific Standard Time)
60427b2277f32877e8fdb052	60427b1e77f32877e8fdb04e	MOVEMENT	0.010558	EXPERIENCE	Fri Mar 05 2021 10:40:34 GMT-0800 (Pacific Standard Time)
60427b2477f32877e8fdb053	60427b1e77f32877e8fdb04e	MOVEMENT	0.031311	EXPERIENCE	Fri Mar 05 2021 10:40:36 GMT-0800 (Pacific Standard Time)
60427b2477f32877e8fdb054	60427b1e77f32877e8fdb04e	VOLUME	0.00055	EXPERIENCE	Fri Mar 05 2021 10:40:36 GMT-0800 (Pacific Standard Time)
60427b2677f32877e8fdb055	60427b1e77f32877e8fdb04e	VOLUME	0.006116	EXPERIENCE	Fri Mar 05 2021 10:40:38 GMT-0800 (Pacific Standard Time)
60427b2677f32877e8fdb056	60427b1e77f32877e8fdb04e	MOVEMENT	0.036473	EXPERIENCE	Fri Mar 05 2021 10:40:38 GMT-0800 (Pacific Standard Time)
60427b2877f32877e8fdb057	60427b1e77f32877e8fdb04e	VOLUME	0.054868	EXPERIENCE	Fri Mar 05 2021 10:40:40 GMT-0800 (Pacific Standard Time)
60427b2877f32877e8fdb058	60427b1e77f32877e8fdb04e	MOVEMENT	0.005466	EXPERIENCE	Fri Mar 05 2021 10:40:40 GMT-0800 (Pacific Standard Time)
60427b2a77f32877e8fdb059	60427b1e77f32877e8fdb04e	MOVEMENT	0.003432	EXPERIENCE	Fri Mar 05 2021 10:40:42 GMT-0800 (Pacific Standard Time)
60427b2a77f32877e8fdb05a	60427b1e77f32877e8fdb04e	VOLUME	0.044183	EXPERIENCE	Fri Mar 05 2021 10:40:42 GMT-0800 (Pacific Standard Time)
60427b2c77f32877e8fdb05b	60427b1e77f32877e8fdb04e	VOLUME	0.036366	EXPERIENCE	Fri Mar 05 2021 10:40:44 GMT-0800 (Pacific Standard Time)
60427b2c77f32877e8fdb05c	60427b1e77f32877e8fdb04e	MOVEMENT	0.011468	EXPERIENCE	Fri Mar 05 2021 10:40:44 GMT-0800 (Pacific Standard Time)

Control/Standard care arm:

Participants will receive standard care monitored over a 4-hour period. Non-pharmacological interventions aimed at distraction such as multi-sensory objects (toys, puzzles, magazines etc) and other audio-visual interventions (music, tablets) will be halted during the trial period. Standard care encapsulates pharmacological and non-pharmacological approaches. These include but may not be limited to use of sedatives, painkillers (opioid and non-opioid) and anti-psychotics or psychotropic medications which will continue to be administered as ordered and at the discretion of the nursing staff in accordance with physician orders and directives recorded on the participants medication administration record. Non-pharmacological approaches such as re-orientation, use of whiteboards, clocks, family presence, repositioning, mobilization and physiotherapy will continue uninterrupted throughout the study period.

Infection Control:

Participants with confirmed or suspected infectious diseases, including but not limited to MRSA (methicillin resistant staphylococcus aureas), C-Diff (clostridium difficile) and SARS-CoV2 (Covid19) will be included in the study. Where this is the case study personnel will follow all local requirements for entering and exiting the patient care areas such as use of masks, gowns, gloves and shields as determined by local infection control regulations. Equipment taken into the patient care area for the purposes of the study will be double cleaned once inside, and once outside the room with the use of disinfectant wipes. The type of wipe used will be determined by nursing and study staff and is dependent on the infectious disease process suspected or confirmed for the participant and in accordance with FHA infection control guidelines.

Study Visits:

The planned duration of the intervention period is 4 hours. Data collected outside of that time frame for the purposes of secondary outcomes will be taken directly from the patient chart and will not require further involvement of the trial participant. In the subsequent iterations of this trial this intervention period will remain consistent

Follow-up Visits:

The intervention is intended to be a single exposure for all planned iterations of the trial. Follow up visits will not be required for the purposes of this trial outside of those timelines described for the access of patient charts to collect data related to the outcomes. A follow up phone call may be made by the research coordinator or designate to ensure continued agreement of participation in the trial after 30 days and in accordance with FHREB and privacy department requirements.

Surveys: Surveys of family members and caregivers are planned. These surveys are intended as a means of assessing the ease of use and acceptability of the intervention, as well as likelihood that parties would willingly use the intervention again if it was offered. Responses will be assessed using a 9-point Likert scale with 4-5 questions. A score of one indicates a strong disagreement, 5-neutral and 9-strong agreement with the questions asked. The surveys also provide a source of feedback for the research team to determine the possible iterative changes for subsequent trial cycles. It is expected that these scores and results will be collated and shared with the research team and industry partner Mindful Garden Digital Health Inc. Surveys will be supplied to family members/ substitute decision makers along with the patient information letter if they are present at or during the interventional study period. Consent language is included in the paper-based form and responses are anonymous. Forms will be collected by the research coordinator or designate and kept in the participant file. Staff members will be provided the survey at completion of the study period and similar consent language is included in the form.

Data Collection**:**

Data collection will be completed by the research coordinator, trainee or research assistant. Standardized CRF's will be used to ensure that the data collected is accurate and that outcome measures are captured throughout the trial period. Please see attached CRF.
Scoring of acuity at baseline will be completed using Apache and SOFA scores which are accepted validated tools. Standardized forms will be used to record the information and then an online calculator (medcalc.com) will be used to calculate the final score which will be entered on to the CRF.
Activity logs, which are password protected will be downloaded periodically to encrypted hard drives for analysis of within participant and within group response to the intervention as well as trends of activity scoring over time.

Both paper and electronic medical records will be accessed during enrollment, the study period and afterwards by the research team to obtain information required for completion of the CRF's and all access to medical records will be conducted in accordance with local regulatory and privacy requirements. Data will be stored in a locked cupboard and will be transcribed on to spreadsheets on encrypted hard drives by the research assistant or designated study team member.

An aim of 10-20 critical care or high acuity patients (divided evenly between control and intervention) will prospectively have additional physiological data collected from the critical care unit central monitoring systems. Patients in these units are routinely monitored with continuous ECG tracings, hemodynamic and respiratory parameters. These systems (Philips brand monitors) store all physiological parameters from individual patient bedsides for a period of at least 24hours continuously in a central monitoring station on each unit so that it can be easily accessed for review purposes. Parameters will be printed off from this central station and de-identified by cutting off the header that may contain patient identifiers and papers
will be relabeled with the unique participant identifier allocated at enrolment.
Parameters include HR, RR, Sa02, MAP and calculated heart rate variability (HRV) analysis including pNN50 (percentage of n-n intervals on an ECG that differ by more than 50ms) and square root of the N-N variance (sqrt N-N variance). Additionally 5 minute periods of ECG tracings will be printed for manual measurement of the R-R intervals (standard measure of heart rate beat to beat measures in milliseconds) and further analysis of HRV prior to study commencement, throughout the study period and in the 2 hours post study completion and similarly de-identified. HRV analysis will not be conducted for patients who have cardiac pacemakers in use, significant cardiac conduction blocks or atrial fibrillation or flutter as these conditions can invalidate the analysis of heart rate variability.

Outcome Measures:

Primary Outcome:

- Agitation (RASS) mean scores over the study period (RASS measured at initiation, Q1H until 1hr post intervention period,
  RASS is defined as the Richmond Agitation and Sedation Scale and is scored from -5 to +4 with -5 meaning unconscious and unresponsive, +4 indicating combative and/or pulling out or attempting to remove lines and tubes (ordinal outcome). Where patients are admitted outside of critical care the scoring for RASS may be conducted by research team members on a Q30min basis to minimize disruption to nursing workload and workflow.

Secondary Outcomes:

- Number of the use of unscheduled (PRN) pharmacological interventions for the management of delirium-associated agitation during the 4-hour study period (Data taken from the medication administration record and nursing flowsheets recorded as a course of standard care). (response-discrete variable)
- Delirium (ICDSC) mean scores over the study period ICDSC at initiation, 2 hrs and 4hrs (study end). ICDSC at beginning of the night shift 1900-1930hrs).
  ICDSC is defined as the Intensive Care Delirium Screening Checklist and is scored from 0-8 with 0 being negative of all aspects of delirium screening and one point added for each category related to screening for

delirium such as disordered thinking, disturbance of day/night routine etc. A score of 4 or greater is considered diagnostic of delirium. (ordinal outcome). Where patients are admitted outside of critical care the scoring for ICDSC may be conducted by research team members to minimize disruption to nursing workload and workflow.

- Activity scores (movement count average and sound input scores decimal score with 0 being the lowest and 1 the highest measured score) (continuous outcome)
- Physiological parameter values and trend over time of the study period including pre and post study (HR, RR, Sa02, MAP and HRV) (continuous outcome)
- Trend in vasopressor use over the study period including pre and post study (continuous outcome)
- Proportion of patients achieving target RASS of 0 (indicating awake and calm). (nominal outcome)
- Use of physical restraints during the 4-hour period and in the 1-hour post intervention conclusion (dichotomous yes/no outcome) and time in mins of restraint presence (continuous outcome).
- Incidence of unplanned removal of intravenous lines, endotracheal tubes or nasogastric tubes by participants throughout the study period (discrete variable) and time to event from start if study period (continuous variable)
- Proportion of participants receiving PRN pharmacological intervention in the 2-hours post intervention. (nominal outcome)
- A 9-point Likert scale-based questionnaire will be administered to bedside family and/or caregivers evaluating ease of use and acceptability of the intervention. Surveys will be provided and collected by the research coordinator or research assistant and will be completed by the caregiver and/ or family member at the conclusion of the study period. A copy of the surveys is included in the appendices. (ordinal data 1=lowest score equates to "strongly disagree" - 9=highest score equates to strongly agree).

Analytical Plan:

Sample Size:

Based on our clinical experience in the ICU, we expect over a period of 4-hours, roughly 70% of enrolled patients will receive PRN (as needed) pharmacological intervention for delirium associated agitation. One trial reported 60-80% PRN antipsychotic use for delirium agitation in a general hospital population, although it is unclear over what time frame this occurred.[43] We assume that the MG intervention will decrease this by a 50% relative reduction from the estimated 70% incidence to 35%. Based on this, the required sample size is 31 patients per arm to achieve a power of 80% with a two tailed comparison at significance level 0.05 increased to n=70 to account for attrition (calculated using an online sample size calculator, www.clinicalc.com). It is difficult to ascertain the true effect size due to a lack of comparable trials in the literature. There is significant heterogeneity amongst outcome reporting in trials including anti-psychotic use or non-pharmacological interventions. Calculations are considered reflective of the research team's experience in the ICU environment and what is considered clinically relevant. For the subsequent iterative trials, it is expected that a smaller sample will be sufficient as this initial trial will provide a more accurate estimate of the treatment effect as well as acting as a control arm for the future iterations.

Statistical Plan:

The primary outcome of agitation scores (RASS) will be described using mean +/-SD and in a linear regression model with the treatment arm as the primary explanatory variable with adjustment for the following baseline patient characteristics: age, ICDSC score, time since last use of pharmacological intervention to calm the patient, sex, presence of underlying brain health condition (eg: dementia, stroke), postoperative or medical cause of admission to the ICU. Incidence of drug administration will be described using median and interquartile range and then further analyzed using a logistic regression model Similarly adjusted models will be used for those receiving infusions of

medications (eg:dexmeditomodine) for the management of delirium associated agitation and vasopressors for hemodynamic management including a delta for change over time for dose of administration throughout the study period. Similarly adjusted linear regression models will be used to assess differences across the two arms in ICDSC. Cox regression models will be used to analyze time-to-event outcomes for unplanned extubation, line removal and nasogastric removal. Survey data will be expressed as mean +/- SD and median and IQR. Mixed methods used to analyze activity scores, descriptive statistics of mean +/- SD, linear regression and repeated measures ANOVA both within patient and within group for the interventional arm.
Physiological measures will be analysed with non-parametric variables expressed as median and IQR with parametric data being expressed as mean +/-SD. Physiological metrics will be analysed along a time continuum for pre study, study start, 1 hour, mid point and study end and 2 hours post with repeated measures testing such as repeated measures ANOVA and Dunnets multiple comparisons tests . Similarly the data that is taken prospectively from the central monitoring system may be collected as frequently as every 1 minute. Where HRV is calculated manually using 5 min ECG strips and R-R intervals the pNN50 and
RMSSD (root mean squared standard deviation) will be calculated and compared between groups.

A-priori subgroup analysis includes patients receiving mechanical ventilation, those with diagnosed delirium >24hours versus new onset (defined as ICDSC≥4), patients in critical care and patients enrolled who have a traumatic brain injury.

We have collaborated with CHEOS (Centre for Health Evaluation and Outcome Sciences) to ensure that the statistical and research plan is fully defined to meet the goals of the trial. CHEOS will also be involved in the statistical analysis between trial periods, will assist in blinded analysis of the outcomes and provide data oversight.

Data will be analyzed at the end of each cycle. As this is an iterative trial design some analysis of the data will be required to determine the variables to be incorporated into the next cycle. Between the second and third cycle of the trial results may determine which visual output is incorporated for testing with the inclusion of an auditory component.

Subject Safety Provisions:

Study progress and patient safety will be regularly evaluated with the help of a Trial Steering Committee aims to meet every 2 weeks for the month prior to the initial trial and after it closes. The trial steering committee will consist of all of the co-applicants listed on this grant application.

Physical Risk:

As the MG screen does not touch or attach to the patient in any way there is minimal to no risk of physical harm to the patient. Positioning of the unit and the mobile nature of the unit should enable straightforward risk mitigation.

Psychological risk:

There is a low risk that exposure to the nature imagery on the platform may adversely increase psycho-motor agitation particularly if the recruited patients have pre-existing phobias to the nature imagery of butterflies and flowers or react to them negatively as hallucinations. Participants exhibiting paranoia may be further agitated by the presence of the camera on the smart TV. As the use of TV's and playing movies is routinely utilized in critical care as a distraction technique, this intervention is considered to be of comparable low risk.

Risk based quality management & patient partner:

Both anticipated and unanticipated risks are likely to occur and having a defined plan of the former will ensure that the latter are minimized. Quality will be constantly managed throughout the various trial stages according to a pre-established plan. With the assistance of a patient partner, the team will balance the risks versus the safety of the study patients and the quality of the trial. The MG device includes a camera and microphone for the detection and

measurement of agitation, these do not have recording capabilities thereby ensuring no video-based data can be accessed outside of the trial period. All trial conduct will adhere to the local privacy and confidentiality requirements and maintain compliance with research ethics boards mandates.

Critical Process and Data Identification:

The following items will be measured and accounted for in reports to the trial steering committee, funding organizations and regulatory bodies. Accrual rate, screening success, drop-outs, recruitment/discontinuation, issue management (number of or type of deviations from the study protocol), data quality (CRF completion), monitoring staff training needs, delegation of responsibilities, calibration of supplies & equipment, DCF's/open queries, percentage of missing critical data, main target parameters, serious adverse events, data for randomization, Compliance with patient selection criteria. These areas will be analyzed in depth and additional areas are likely to be determined and expanded on.

Risk Identification:

Potential risks for the *study participants* are unexpected adverse events, serious adverse events (expected and unexpected) and potential loss of confidentiality. Potential risks for the *validity of the data* are the inclusion of inappropriate patients, incomplete data and protocol deviations. Audits of data collection and completion of CRF's will take place according to a pre-determined schedule of every 5th recruited participant by the trainee to ensure accuracy of the data, transcription and adherence to study protocols.

Risk Evaluation:

The likelihood of resulting damage is difficult to define at this time but assumed to be low.

Risk Control:

Proper education and oversight should minimize risks, but potential risks might not be mitigated. A clinical phase monitoring plan ensures that the Clinical Research Associate (CRA) will have detailed instructions on how he/she should respond to risk indicators. A case by case plan for the training of research assistants will be implemented according to prior experience with a minimum of two hours with the research coordinator and trainee prior to assuming duties of data collection. Changes to the monitoring approach, will occur based on findings such as those described above.

Risk Communication:

The clinical monitoring plan will document the projected risks and any findings within or outside the scope will be addressed during team meetings. This will ensure that assigned risks, as well as potential unforeseen new ones, are addressed in a timely and appropriate manner. Positive or negative outcomes will be documented to refer back to previously collected information ensuring transparency, understanding and process execution resulting in smoother study conduct.

Risk Review:

A continuous risk review will occur and adapted as new risks arise, previously defined ones are deemed less relevant or if others rise in importance. Continuous review will become relevant during the time of study design, conduct, study close out, post study data analysis and final report.

Risk Reporting:

All stakeholders will be apprised of the risk management activities in routine reports. Any deviations outside the predefined tolerance limit will be clarified and the Clinical Trial Quality Management Plan approach described.

Serious Adverse Event Reporting:

Unexpected or adverse findings will be recorded in progress notes in the participant file by the clinical research coordinator or designate. The research coordinator will screen the participants medical record during data retrieval on the following day after study completion, or on the following weekday where the intervention period occurs on a Friday. As well as screening the patient medical record the research coordinator will communicate with the unit care team to determine any possible adverse events requiring reporting. Guidelines for Good Clinical Practice will be followed in accordance with the FHA Guidance Notes for Reporting Unanticipated Problems to the FHREB. A copy of the guidelines are included in the RISe documents section 9.8. Where serious adverse events are found and meet the criteria as Medical Device Serious Adverse Events and Unanticipated Problems, reporting to the FHREB will be made no later than 7 days of the event using the "Request for Acknowledgement Form" provided by FHREB

Circumstances under which the trial would be stopped early:

Decisions to stop the trial early will be made by the PI in collaboration with the Trial Steering Committee and reported to all regulatory boards and funding organizations in accordance with required reporting procedures. Reasons for early discontinuation include but may not be limited to:

- Excessively slow recruitment
- Study determined by the PI and Trial Steering Committee to be futile in regard to achieving the aims as guided by the data analysis
- Determination of unexpected, significant, or unacceptable risk to patients
- Inadequate adherence to protocol requirements
- Inadequate complete and/or evaluable data
- Non-compliance with the International Conference on Harmonization (ICH) guideline for Good Clinical Practice (GCP)
- No further study activity (i.e., all patients have completed, and all obligations have been fulfilled)

Ethical Considerations:

Potential Benefits:

The use of pharmacological agents in the treatment and management of delirium carry risks such as over-sedation, QT prolongation (measure of electrical heart activity associated with an increased risk of dangerous cardiac arrythmia) and adverse drug interactions. A reduction in reliance on these medications may in turn lower the risk profile to the patient and improve overall patient outcomes. Similarly, lowering the reliance on physical restraints may also reduce adverse events related to increased agitation, self extubation and mental distress. It is possible that there may be a reduction in the severity and length of delirium symptoms which could translate to better overall outcomes as well as reduced ICU and hospital length of stay.

Potential Risks:

The Mindful Garden platform is considered a Class 1 medical device in accordance with rule 12 of Health Canada device classification "Any other active device is classified as Cass I" and confirmed by the senior Regulatory Affairs Officer of Health Canada on July 14th 2020 as not requiring an ITA (investigational testing authorization). This

denotes MG as a low risk intervention. As Mindful Garden does not touch or attach to the patient in any way there is minimal to no risk of physical harm to the patient. There is a low risk that exposure to the nature imagery on the platform may adversely increase psycho-motor agitation particularly if the recruited patients have pre-existing phobias to the nature imagery of butterflies and flowers or react to them negatively as hallucinations. As the use of TV's and playing movies is routinely utilized in the ICU/HAU at RCH, this is considered by the PI's to be of comparable low risk as current standard management strategies.

Ethics Approval**:**

This protocol will be submitted through the RISe UBC platform for harmonized REB review with both the Fraser Health Authority and Simon Fraser University. All study conduct will be in accordance with FHREB regulations and guidelines.

Plans for Publication and conference presentations:

It is expected that the results of this study will be generate at least 2-3 podium or poster presentations at conferences as well as publication in a critical care journal. The study and results will also be used for completion of the thesis and doctoral study requirements for trainee Michelle Nicholas under the supervision of PI Dr Steven Reynolds.

REFERENCES:

1. Inouye SK, Rushing JT, Foreman MD, Palmer RM, Pompei P. Does delirium contribute to poor hospital outcomes? A three-site epidemiologic study. *Journal of General Internal Medicine*. 1998;13(4):234-242. doi:10.1046/j.1525-1497.1998.00073.x

2. Kalabalik J, Brunetti L, El-Srougy R. Intensive care unit delirium: a review of the literature. *J Pharm Pract*. 2014;27(2):195-207. doi:10.1177/0897190013513804

3. Meagher D, O'Regan N, Ryan D, et al. Frequency of delirium and subsyndromal delirium in an adult acute hospital population. *Br J Psychiatry*. 2014;205(6):478-485. doi:10.1192/bjp.bp.113.139865

4. Leslie DL. One-Year Health Care Costs Associated With Delirium in the Elderly Population. *Arch Intern Med*. 2008;168(1):27. doi:10.1001/archinternmed.2007.4

5. Leslie DL, Inouye SK. The Importance of Delirium: Economic and Societal Costs. *J Am Geriatr Soc*. 2011;59(Suppl 2):S241-S243. doi:10.1111/j.1532-5415.2011.03671.x

6. Devlin JW, Skrobik Y, Gelinas C, et al. Clinical Practice Guidelines for the Prevention and Management of Pain, Agitation/Sedation, Delirium, Immobility, and Sleep Disruption in Adult Patients in the ICU. *Crit Care Med*. 2018;46(9):E825-E873. doi:10.1097/CCM.0000000000003299

7. Giusti GD, Piergentili F. ICU delirium, is there a correct management strategy? *Australian Critical Care*. 2012;25(3):153-154. doi:10.1016/j.aucc.2012.05.002

8. Zoremba N. Management of delirium in the intensive care unit. Non-pharmacological therapy options. *Med Klin-Intensivmed Notfallmed*. 2017;112(4):320-325. doi:10.1007/s00063-015-0123-y

9. Ely EW. The ABCDEF Bundle: Science and Philosophy of How ICU Liberation Serves Patients and Families. *Crit Care Med*. 2017;45(2):321-330. doi:10.1097/CCM.0000000000002175

10. Rose L, Burry L, Mallick R, et al. Prevalence, risk factors, and outcomes associated with physical restraint use in mechanically ventilated adults. *J Crit Care*. 2016;31(1):31-35. doi:10.1016/j.jcrc.2015.09.011

11. Azizpour M, Moosazadeh M, Esmaeili R. Use of Physical Restraints in Intensive Care Unit: A Systematic Review Study. *Acta Medica Mediterr*. 2017;33(1):129-136. doi:10.19193/0393-6384_2017_1_020

12. Luk E, Sneyers B, Rose L, et al. Predictors of physical restraint use in Canadian intensive care units. *Crit Care*. 2014;18(2):R46. doi:10.1186/cc13789

13. Benbenbishty J, Adam S, Endacott R. Physical restraint use in intensive care units across Europe: The PRICE study. *Intensive and Critical Care Nursing*. 2010;26(5):241-245. doi:10.1016/j.iccn.2010.08.003

14. Chang L, Wang KK, Chao Y. Influence of physical restraint on unplanned extubation of adult intensive care patients: a case-control study. *American Journal of Critical Care*. 2008;17(5):408-416.

15. Curry K, Cobb S, Kutash M, Diggs C. Characteristics associated with unplanned extubations in a surgical intensive care unit. *American Journal of Critical Care*. 2008;17(1):45-52.

16. Schmitt EM, Gallagher J, Albuquerque A, et al. Perspectives on the Delirium Experience and Its Burden: Common Themes Among Older Patients, Their Family Caregivers, and Nurses. *The Gerontologist*. 2019;59(2):327-337. doi:10.1093/geront/gnx153

17. Neufeld KJ, Yue J, Robinson TN, Inouye SK, Needham DM. Antipsychotic Medication for Prevention and Treatment of Delirium in Hospitalized Adults: A Systematic Review and Meta-Analysis. *J Am Geriatr Soc*. 2016;64(4):705-714. doi:10.1111/jgs.14076

18. Bak M, Weltens I, Bervoets C, et al. The pharmacological management of agitated and aggressive behaviour: A systematic review and meta-analysis. *Eur Psychiat*. 2019;57:78-100. doi:10.1016/j.eurpsy.2019.01.014

19. Flaherty JH, Gonzales JP, Dong B. Antipsychotics in the Treatment of Delirium in Older Hospitalized Adults: A Systematic Review. *Journal of the American Geriatrics Society*. 2011;59(s2):S269-S276. doi:10.1111/j.1532-5415.2011.03675.x

20. Marra A, Ely EW, Pandharipande PP, Patel MB. The ABCDEF Bundle in Critical Care. *Crit Care Clin*. 2017;33(2):225-243. doi:10.1016/j.ccc.2016.12.005

21. Collet MO, Caballero J, Sonneville R, et al. Prevalence and risk factors related to haloperidol use for delirium in adult intensive care patients: the multinational AID-ICU inception cohort study. *Intensive Care Med*. 2018;44(7):1081-1089. doi:10.1007/s00134-018-5204-y

22. Inouye SK, Marcantonio ER, Metzger ED. Doing damage in delirium: the hazards of antipsychotic treatment in elderly people. *Lancet Psychiatry*. 2014;1(4):312-315. doi:10.1016/S2215-0366(14)70263-9

23. Bakri MH, Ismail EA, Ibrahim A. Comparison of dexmedetomidine or ondansetron with haloperidol for treatment of postoperative delirium in trauma patients admitted to intensive care unit: randomized controlled trial. *Anaesth Pain Intensive Care*. 2015;19(2):118-123.

24. Tan JA, Ho KM. Use of dexmedetomidine as a sedative and analgesic agent in critically ill adult patients: a meta-analysis. *Intensive Care Med*. 2010;36(6):926-939. doi:10.1007/s00134-010-1877-6

25. Cooke ML, Moyle W, Shum DHK, Harrison SD, Murfield JE. A randomized controlled trial exploring the effect of music on agitated behaviours and anxiety in older people with dementia. *Aging & Mental Health*. 2010;14(8):905-916. doi:10.1080/13607861003713190

26. Johnson K, Fleury J, McClain D. Music intervention to prevent delirium among older patients admitted to a trauma intensive care unit and a trauma orthopaedic unit. *Intensive Crit Care Nurs*. 2018;47:7-14. doi:10.1016/j.iccn.2018.03.007

27. Bannon L, McGaughey J, Verghis R, Clarke M, McAuley DF, Blackwood B. The effectiveness of non-pharmacological interventions in reducing the incidence and duration of delirium in critically ill patients: a systematic review and meta-analysis. *Intensive Care Med*. 2019;45(1):1-12. doi:10.1007/s00134-018-5452-x

28. Atzori B, Hoffman HG, Vagnoli L, Messeri A, Grotto RL. Virtual Reality as Distraction Technique for Pain Management in Children and Adolescents. *Advanced Methodologies and Technologies in Medicine and Healthcare*. Published online 2019:483-494. doi:10.4018/978-1-5225-7489-7.ch038

29. Gold JI, Kim SH, Kant AJ, Joseph MH, Rizzo A. Effectiveness of virtual reality for pediatric pain distraction during. *Cyberpsychology & Behavior*. 2006;9(2):207-212. doi:10.1089/cpb.2006.9.207

30. Malloy KM, Milling LS. The effectiveness of virtual reality distraction for pain reduction: A systematic review. *Clinical Psychology Review*. 2010;30(8):1011-1018. doi:10.1016/j.cpr.2010.07.001

31. Gupta A, Scott K, Dukewich M. Innovative Technology Using Virtual Reality in the Treatment of Pain: Does It Reduce Pain via Distraction, or Is There More to It? *Pain Med*. 2018;19(1):151-159. doi:10.1093/pm/pnx109

32. Turon M, Fernandez-Gonzalo S, Jodar M, et al. Feasibility and safety of virtual-reality-based early neurocognitive stimulation in critically ill patients. *Ann Intensive Care*. 2017;7. doi:10.1186/s13613-017-0303-4

33. Ohly H, White MP, Wheeler BW, et al. Attention Restoration Theory: A systematic review of the attention restoration potential of exposure to natural environments. *J Toxicol Env Health-Pt b-Crit Rev*. 2016;19(7):305-343. doi:10.1080/10937404.2016.1196155

34. Ulrich R, Simons R, Losito B, Fiorito E, Miles M, Zelson M. Stress Recovery During Exposure to Natural and Urban Environments. *J Environ Psychol*. 1991;11(3):201-230. doi:10.1016/S0272-4944(05)80184-7

35. Gerber SM, Jeitziner MM, Wyss P, et al. Visuo-acoustic stimulation that helps you to relax: A virtual reality setup for patients in the intensive care unit. *Sci Rep*. 2017;7. doi:10.1038/s41598-017-13153-1

36. Gold JI, Mahrer NE. Is Virtual Reality Ready for Prime Time in the Medical Space? A Randomized Control Trial of Pediatric Virtual Reality for Acute Procedural Pain Management. *Journal of Pediatric Psychology*. 2018;43(3):266-275. doi:10.1093/jpepsy/jsx129

37. Guttman, Gloria. Use of the Digital Therapeutic Intervention Mindful Garden for de-escalation of behavioural symptoms of dementia. Published online 2020.

38. Sessler CN, Gosnell MS, Grap MJ, et al. The Richmond Agitation–Sedation Scale. *Am J Respir Crit Care Med*. 2002;166(10):1338-1344. doi:10.1164/rccm.2107138

39. Bergeron N, Dubois MJ, Dumont M, Dial S, Skrobik Y. Intensive Care Delirium Screening Checklist: evaluation of a new screening tool. *Intensive Care Med*. 2001;27(5):859-864. doi:10.1007/s001340100909

40. Ibrahim Khalil, McCarthy Cian P., McCarthy Killian J., et al. Delirium in the Cardiac Intensive Care Unit. *Journal of the American Heart Association*. 7(4):e008568. doi:10.1161/JAHA.118.008568

41. Zywiel MG, Hurley RT, Perruccio AV, Hancock-Howard RL, Coyte PC, Rampersaud YR. Health Economic Implications of Perioperative Delirium in Older Patients After Surgery for a Fragility Hip Fracture: *The Journal of Bone and Joint Surgery*. 2015;97(10):829-836. doi:10.2106/JBJS.N.00724

42. Inouye SK, van Dyck CH, Alessi CA, Balkin S, Siegal AP, Horwitz RI. Clarifying Confusion: The Confusion Assessment Method. *Ann Intern Med*. 1990;113(12):941-948. doi:10.7326/0003-4819-113-12-941

43. Pinkhasov A, James SA, Fazzari M, Singh D, Lam S. Role of Ramelteon in Reduction of As-Needed Antipsychotics in Elderly Patients with Delirium in a General Hospital Setting. *Clin Drug Investig*. 2017;37(12):1137-1141. doi:10.1007/s40261-017-0573-5
