# Supplementary material for: A randomized, clinical trial investigating the use of a digital intervention to reduce delirium-associated agitation
Source: NPJ Digit Med. 2023 Oct 30;6:202. doi: 10.1038/s41746-023-00950-4 (PMC10616287; doi:10.1038/s41746-023-00950-4)
Supplement: Supplementary file 2 — Reporting Summary [file 41746_2023_950_MOESM2_ESM.pdf]

nature portfolio

Corresponding author(s): Dr. Steven Reynolds

Last updated by author(s): 2023-SEP-29

Reporting Summary

Nature Portfolio wishes to improve the reproducibility of the work that we publish. This form provides structure for consistency and transparency in reporting. For further information on Nature Portfolio policies, see our Editorial Policies and the Editorial Policy Checklist.

Statistics

For all statistical analyses, confirm that the following items are present in the figure legend, table legend, main text, or Methods section.

n/a	Confirmed	
☐	☒	The exact sample size (*n*) for each experimental group/condition, given as a discrete number and unit of measurement
☐	☒	A statement on whether measurements were taken from distinct samples or whether the same sample was measured repeatedly
☐	☒	The statistical test(s) used AND whether they are one- or two-sided<br>*Only common tests should be described solely by name; describe more complex techniques in the Methods section.*
☐	☒	A description of all covariates tested
☒	☐	A description of any assumptions or corrections, such as tests of normality and adjustment for multiple comparisons
☐	☒	A full description of the statistical parameters including central tendency (e.g. means) or other basic estimates (e.g. regression coefficient) AND variation (e.g. standard deviation) or associated estimates of uncertainty (e.g. confidence intervals)
☐	☒	For null hypothesis testing, the test statistic (e.g. *F*, *t*, *r*) with confidence intervals, effect sizes, degrees of freedom and *P* value noted<br>*Give P values as exact values whenever suitable.*
☒	☐	For Bayesian analysis, information on the choice of priors and Markov chain Monte Carlo settings
☒	☐	For hierarchical and complex designs, identification of the appropriate level for tests and full reporting of outcomes
☒	☐	Estimates of effect sizes (e.g. Cohen's *d*, Pearson's *r*), indicating how they were calculated

Our web collection on statistics for biologists contains articles on many of the points above.

Software and code

Policy information about availability of computer code

Data collection	Data was collected via a combination of paper case report forms as well as Microsoft Excel for Mac version 16.77.1
Data analysis	The main statistical analysis for the outcomes of RASS, regression and subgroup analyses were conducted by an independent statistician using SAS Version 9.1. Secondary outcomes were analyzed using GraphPad Prism Version 9.4.1.

For manuscripts utilizing custom algorithms or software that are central to the research but not yet described in published literature, software must be made available to editors and reviewers. We strongly encourage code deposition in a community repository (e.g. GitHub). See the Nature Portfolio guidelines for submitting code & software for further information.

Data

Policy information about availability of data

All manuscripts must include a data availability statement. This statement should provide the following information, where applicable:

- Accession codes, unique identifiers, or web links for publicly available datasets
- A description of any restrictions on data availability
- For clinical datasets or third party data, please ensure that the statement adheres to our policy

Aggregate data analyzed in this study may be made available upon reasonable request by contacting the corresponding author via the e-mail address provided.

Research involving human participants, their data, or biological material

Policy information about studies with human participants or human data. See also policy information about sex, gender (identity/presentation), and sexual orientation and race, ethnicity and racism.

Reporting on sex and gender	Participant sex was collected via information available on their patient chart and relevant medical records. This study collected information about patient sex only, data is not currently presented stratified by sex. As this was the first clinical trial of this type of intervention, data collection was exploratory and general in nature, this study was not designed to assess differences in response to this intervention based on sex and gender. A larger, multi-site RCT is planned for the future with a focus on GBA+ analysis.
Reporting on race, ethnicity, or other socially relevant groupings	As this was the first clinical trial of this type of intervention, data collection was exploratory and general in nature, this study was not designed to assess differences in response to this intervention based on race, ethnicity, or other socially-relevant factors. A larger, multi-site RCT is planned for the future with a focus on EDI analysis.
Population characteristics	Information about age, sex, BMI, as well as % of patients with: renal replacement therapy, COPD, underlying brain health condition (TBI, stroke, dementia), psychiatric history (depression, anxiety disorder, bi-polar), substance use history, COVID-19 positive, diabetic, and who were mechanically ventilated was collected. We also collected information about days since first delirium diagnosis, APACHE IV score, and admission diagnosis. This data is presented in Table 1.
Recruitment	Participants were recruited from hospital wards and ICU based on screening for inclusion/exclusion criteria by members of the research team. There was no self-selection that could have led to bias.
Ethics oversight	Harmonized ethics approval was obtained from Fraser Health Authority and Simon Fraser University regulatory ethics boards.

Note that full information on the approval of the study protocol must also be provided in the manuscript.

Field-specific reporting

Please select the one below that is the best fit for your research. If you are not sure, read the appropriate sections before making your selection.

☒ Life sciences ☐ Behavioural & social sciences ☐ Ecological, evolutionary & environmental sciences

For a reference copy of the document with all sections, see nature.com/documents/nr-reporting-summary-flat.pdf

Life sciences study design

All studies must disclose on these points even when the disclosure is negative.

Sample size	Based on clinical experience in the ICU, it was anticipated that over a period of 4 hours, approximately 70% of agitated delirious patients would receive unscheduled medications for delirium. We anticipated the intervention would decrease this by a 50% relative reduction from 70% incidence to 35%. The required sample size was calculated to be 31 patients per arm, with a power of 80% and a significance level of 0.05. (www.clinicalc.com) We increased this slightly in recognition that it was an estimated effect size and is supported by previous literature
Data exclusions	No data was excluded from the analysis.
Replication	Findings were not reproduced due to budgetary and time constraints of completing the study. A future, larger trial is planned with more participants and greater number of data collected from participants.
Randomization	Eligible patients were randomized using a master randomization list generated by an independent statistician using block permutation (blocks of 2 or 4). Allocation was determined using sequentially numbered opaque envelopes previously filled by a non-research team member and opened after enrollment was confirmed.
Blinding	Blinding to the intervention was not possible due to the nature of the intervention and the logistical constraints of the study.

Reporting for specific materials, systems and methods

We require information from authors about some types of materials, experimental systems and methods used in many studies. Here, indicate whether each material, system or method listed is relevant to your study. If you are not sure if a list item applies to your research, read the appropriate section before selecting a response.

Materials & experimental systems

n/a	Involved in the study
☒	☐ Antibodies
☒	☐ Eukaryotic cell lines
☒	☐ Palaeontology and archaeology
☒	☐ Animals and other organisms
☐	☒ Clinical data
☒	☐ Dual use research of concern
☒	☐ Plants

Methods

n/a	Involved in the study
☒	☐ ChIP-seq
☒	☐ Flow cytometry
☒	☐ MRI-based neuroimaging

Clinical data

Policy information about clinical studies

All manuscripts should comply with the ICMJE guidelines for publication of clinical research and a completed CONSORT checklist must be included with all submissions.

Clinical trial registration

This study was registered with ClinicalTrials.gov, NCT04652622.

Study protocol

Full trial protocol is provided in the Supplementary Materials document submitted alongside the manuscript.

Data collection

We conducted a single-center, open-label randomized controlled trial at a tertiary referral and trauma center (Royal Columbian Hospital) in New Westminster, Canada. The study continued until recruitment goals were met. Participants had to be admitted to intensive care, high acuity, and cardiac telemetry units. Eligible patients were randomized in a 1:1 ratio to either intervention plus standard of care, or standard of care only. A total of 73 participants were recruited between March 16th, 2021 and January 5th, 2022, with 70 included in the final analysis (See Fig 1.0). Three participants were excluded after randomization, of these two before the study start due to changes to the course of clinical care, and one that was a duplicate enrolment. Participants were recruited from critical care (n=65) and high acuity cardiac telemetry wards (n=5). See Table 1 for further details on patient demographics and characteristics.

Outcomes

The primary outcome was mean agitation (RASS) scores over the study period. RASS scores were measured pre-exposure, and every hour thereafter until one hour post the 4-hour intervention period. Secondary outcomes included the proportion of participants receiving unscheduled pharmacological interventions for the management of delirium-associated agitation during the 4-hour study period, delirium scores (ICDSC at intervention initiation, 2hrs, and 4hrs exposure time), the proportion of patients achieving target RASS of 0 or -1 (indicating awake and calm to mildly drowsy), the use of physical restraints, the incidence of self-inflicted, unplanned removal of lines, tubes or equipment by participants throughout the study period, including the time to event from the start of the study period of these events, as well as the proportion of participants receiving unscheduled pharmacological intervention in the 2-hours post-intervention. Information required to collect primary and secondary outcomes was found by reviewing patient charts and medication administration records. Delirium and agitation scores were assigned either based on evaluation by the patients treating nurse (in the ICU), pr by members of the research team (in the ward setting).
